# Mapping the Conformational Landscape of the Neutral Network of RNA Sequences That Connect Two Functional Distinctly Different Ribozymes

**DOI:** 10.1002/cbic.202200022

**Published:** 2022-02-15

**Authors:** Bozana Knezic, Sara Keyhani‐Goldau, Harald Schwalbe

**Affiliations:** ^1^ Institute for Organic Chemistry and Chemical Biology Center for Biomolecular Magnetic Resonance (BMRZ) Johann Wolfgang Goethe University Max-von-Laue-Str. 7 60438 Frankfurt/Main Germany

**Keywords:** analytical chemistry, NMR spectroscopy, ribozymes, RNA structures, SHAPE analysis

## Abstract

During evolution of an RNA world, the development of enzymatic function was essential. Such enzymatic function was linked to RNA sequences capable of adopting specific RNA folds that possess catalytic pockets to promote catalysis. Within this primordial RNA world, initially evolved self‐replicating ribozymes presumably mutated to ribozymes with new functions. Schultes and Bartel (*Science* **2000**, *289*, 448–452) investigated such conversion from one ribozyme to a new ribozyme with distinctly different catalytic functions. Within a neutral network that linked these two prototype ribozymes, a single RNA chain could be identified that exhibited both enzymatic functions. As commented by Schultes and Bartel, this system possessing one sequence with two enzymatic functions serves as a paradigm for an evolutionary system that allows neutral drifts by stepwise mutation from one ribozyme into a different ribozyme without loss of intermittent function. Here, we investigated this complex functional diversification of ancestral ribozymes by analyzing several RNA sequences within this neutral network between two ribozymes with class III ligase activity and with self‐cleavage reactivity. We utilized rapid RNA sample preparation for NMR spectroscopic studies together with SHAPE analysis and in‐line probing to characterize secondary structure changes within the neutral network. Our investigations allowed delineation of the secondary structure space and by comparison with the previously determined catalytic function allowed correlation of the structure‐function relation of ribozyme function in this neutral network.

## Graphical abstract

**The diversity** of secondary structures of different ribozymes connected through several mutational steps were investigated. We mapped the mutational landscape in order to understand changes in secondary structure and their link to particular functions. This work further highlights the necessity for the utilization of different complementary techniques for the analysis of RNA secondary structures.

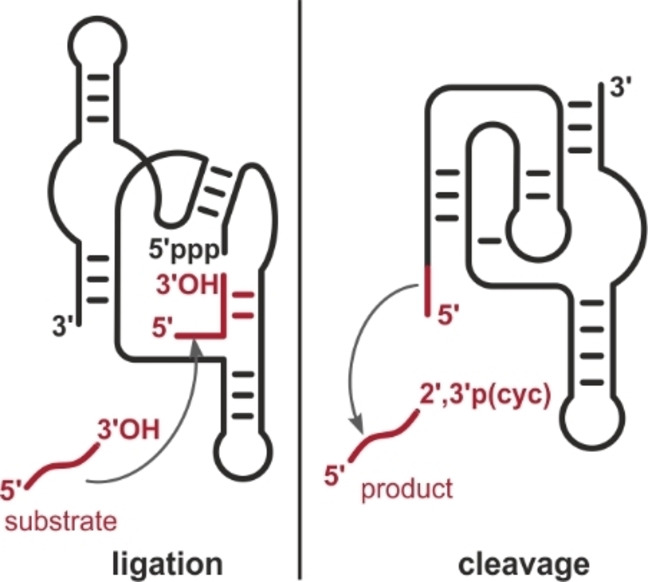

## Introduction

**Figure 1:**
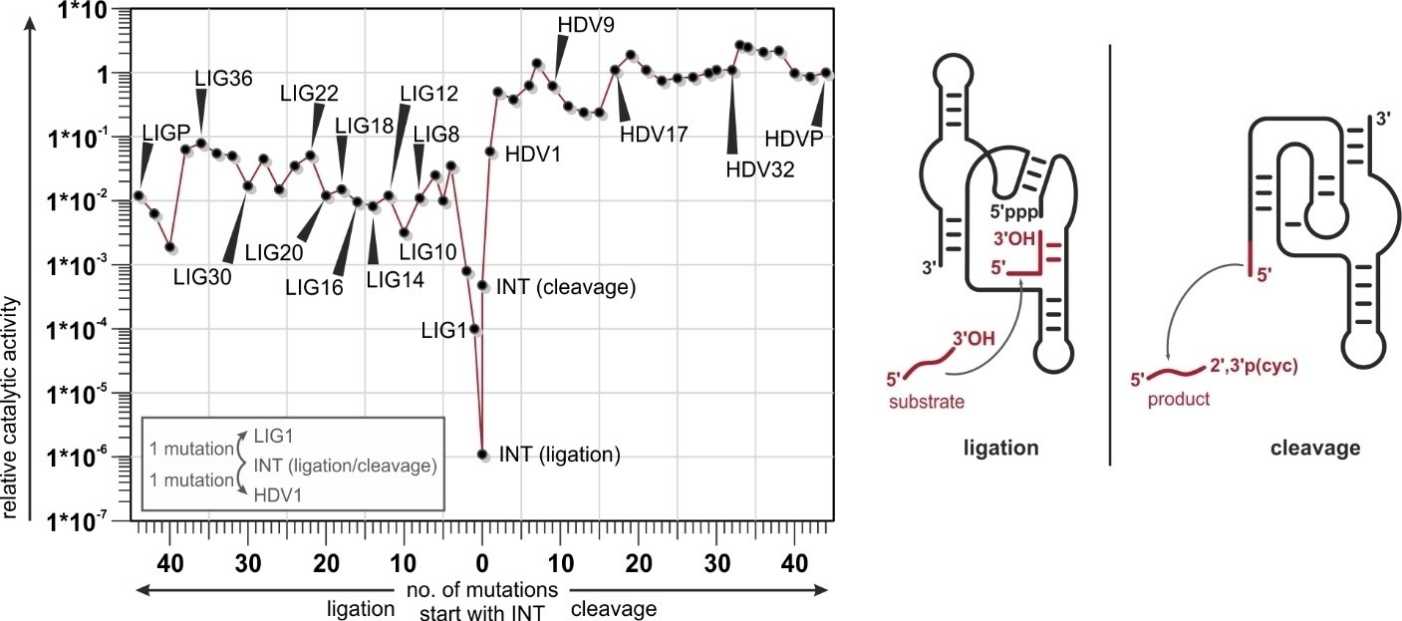
Ligase and cleavage activities are shown for all constructs from the neutral network. The numeral indicates the number of mutations relative to the sequence of the INT construct as shown below the graph. In addition a legend explains the numbering. Catalytic activities are normalized relative to HDVP activity. The figure was adopted from Schultes and Bartel^[13]^ and rearranged. Catalyzed reactions are shown with secondary structures of ligase ribozyme and HDV ribozyme. Ligated substrate and released product are highlighted in red. Ligation with ligase ribozyme occurs in presence of substrate RNA with a free accessible 3’OH group. Cleavage results in a released product RNA with a 2’,3’ cyclic phosphate. INT catalyzes both reactions.

Today's broadly accepted hypothesis of the evolution of life assumes the existence of an RNA‐world,[^1^, ^2^, ^3^] strongly supported by the discovery of ribozymes,[^4^, ^5^, ^6^, ^7^, ^8^] catalytically active RNAs present in all kingdoms of life. Within a prebiotic system, initially evolved ribozymes inevitably underwent numerous mutations^[9]^ to improve catalytic efficacy, but also to diversify catalytic function.[^9^, ^10^, ^11^, ^12^] To understand evolutionary requirements for change of ancestral species, we here characterize a so‐called neutral mutational RNA network (Figure 1) originally investigated by Schultes and Bartel.^[13]^ At the endpoints of this neutral mutational network of RNA are two ribozymes, a class III self‐ligating ligase composed of 87 nucleotides (nts) (including a substrate of 9 nts)^[14]^ and an HDV self‐cleaving ribozyme of 86 nts (including a product of 8 nts).^[15]^ The two RNAs with opposing catalytic properties do not share any apparent structural elements. Schultes and Bartel designed a neutral network, in which one ribozyme is converted into the other by mutating sequences in multiple steps. For an 87 nucleotide‐long RNA sequence, there are 4^87^ = 2×10^52^ different sequences, but any one sequence can be converted into any other sequence within this astronomically large sequence space by not more than 88 mutations.

Schultes and Bartel investigated a neutral network composed of 51 RNA sequences that links the two prototype ligase and cleavage ribozymes by stepwise introducing mutations, most often single point mutations. Interestingly, a single intersection sequence was found that possessed both functions and was called two‐function ribozyme (Figure 1). Since this network features both a conformational and a functional landscape, we investigated secondary structure changes along the neutral network. We applied chemical probing methodology (SHAPE and in‐line probing) together with complementary 1D‐ and 2D‐liquid‐state NMR spectroscopy[^16^, ^17^, ^18^] to derive RNA secondary structures. We show that changes in the ground state secondary structure do not strictly correlate with the catalytic reactivity changes within this neutral network, suggesting the involvement of low‐populated high energy states for catalytic activity.

## Results and Discussion

**Figure 2:**
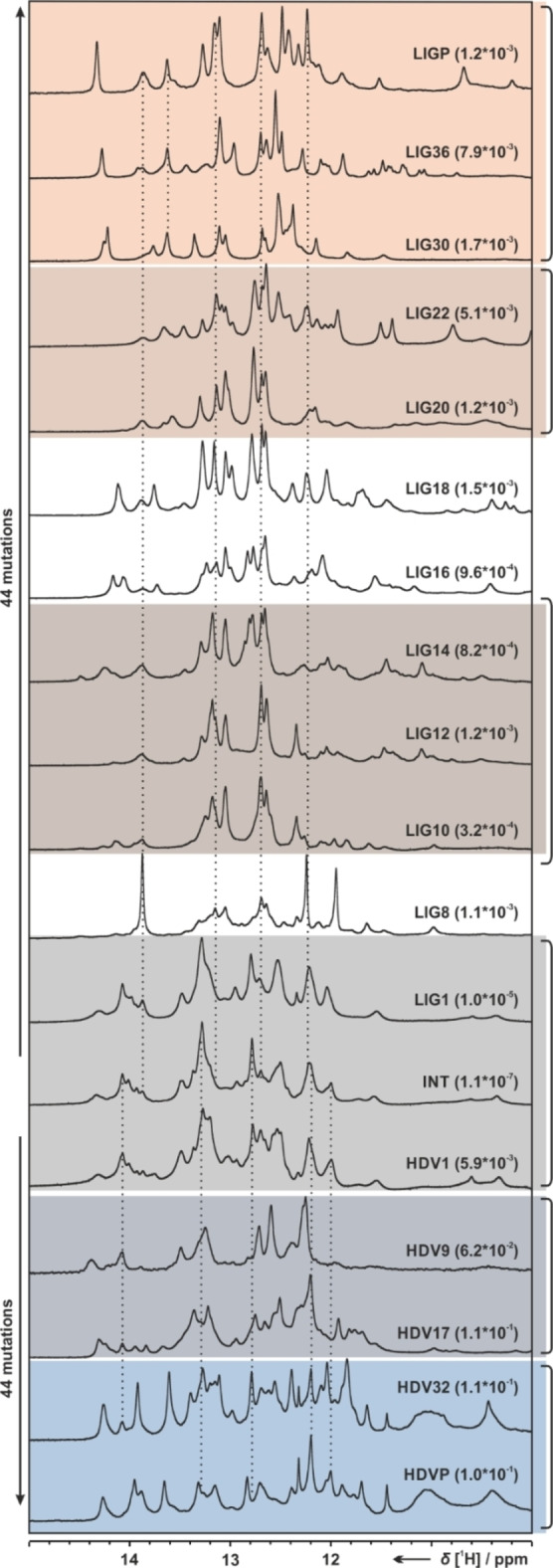
Imino regions of 1D ^1^H NMR spectra of eighteen different members of the neutral network (LIGP, LIG36, LIG30, LIG22, LIG20, LIG18, LIG16, LIG14, LIG12, LIG10, LIG8, LIG1, INT, HDV1, HDV9, HDV17, HDV32, HDVP) are shown. Dashed lines indicate similarities assigned to secondary structure elements as outlined later in Figure 4. Spectra were recorded at 600–950 MHz and 298 K, with 1536 scans and with 25 mM potassium phosphate buffer and 10 % D2O using an NMR jump‐and‐return echo pulse sequence for water suppression. Sample concentrations were 100–200 μM. Ribozymes with similar secondary structures are clustered by colors.

Figure 2 shows selected regions of 1D ^1^H spectra^[19]^ of RNAs, twelve individual RNAs with ligase catalytic activity (LIG1‐LIG36), the prototype ligase RNA (LIGP), five RNAs with self‐cleavage catalytic activity (HDV1‐HDV32) and the prototype cleavage RNA (HDVP) together with the intersection RNA (INT). The numeral indicates the number of mutations relative to the sequence of the INT sequence, known as the Shannon distance. The selected regions in the 1D NMR spectra show the fingerprint region of imino hydrogen atoms that are protected from solvent exchange through involvement in stable secondary structure, most often due to hydrogen bond formation.

Analysis of this fingerprint region indicates significant changes in the secondary structure for the different RNAs. Both prototype sequences LIGP and HDVP feature signatures of stable, rigid secondary structure: This stable, non‐dynamic secondary structure is retained for closely related sequences. The presence of the stable secondary structure can be deduced from the observation of sharp imino proton signals. The number of detectable signals decreases for sequences between LIG36 and LIG30 as well as between HDV32 and HDV17. The intersecting sequences INT and closely related sequences feature broad NMR fingerprints. Such NMR spectra with broad signals indicate the existence of multiple RNA conformations in intermediate exchange and increased exchange of imino protons with solvent water. The findings are in accordance with the original publication where INT was reported to adopt both, ligase and HDV secondary structures featuring two distinct populations but not a single “combined” secondary structure of those. Furthermore, spectra of LIG1 and HDV1, a single mutation away from INT, are very similar to the INT spectrum revealing related secondary structure populations even though the activities of LIG1 increases 90 times (1×10^−4^) and for HDV1 (5.9×10^−3^) increases 120 times (Figure 1). Surprisingly, HDV9 and HDV17, with eight nucleotides difference in their sequences, show similar spectra.

Sequences with a larger number of mutations show large differences, sometimes even for neighboring sequences, revealing that large conformational changes can occur by a single or two mutations. The sequences of LIG8 and LIG10 or LIG18 and LIG20 vary by two nucleobases but the NMR 1D ^1^H signal pattern in the imino region are entirely different indicating a complete different fold. As shown later, reappearing signals (dashed line in Figure 2) were assigned to individual imino protons (Figure 4) and show secondary structure elements that remain stable throughout the entire neutral network.

**Figure 3:**
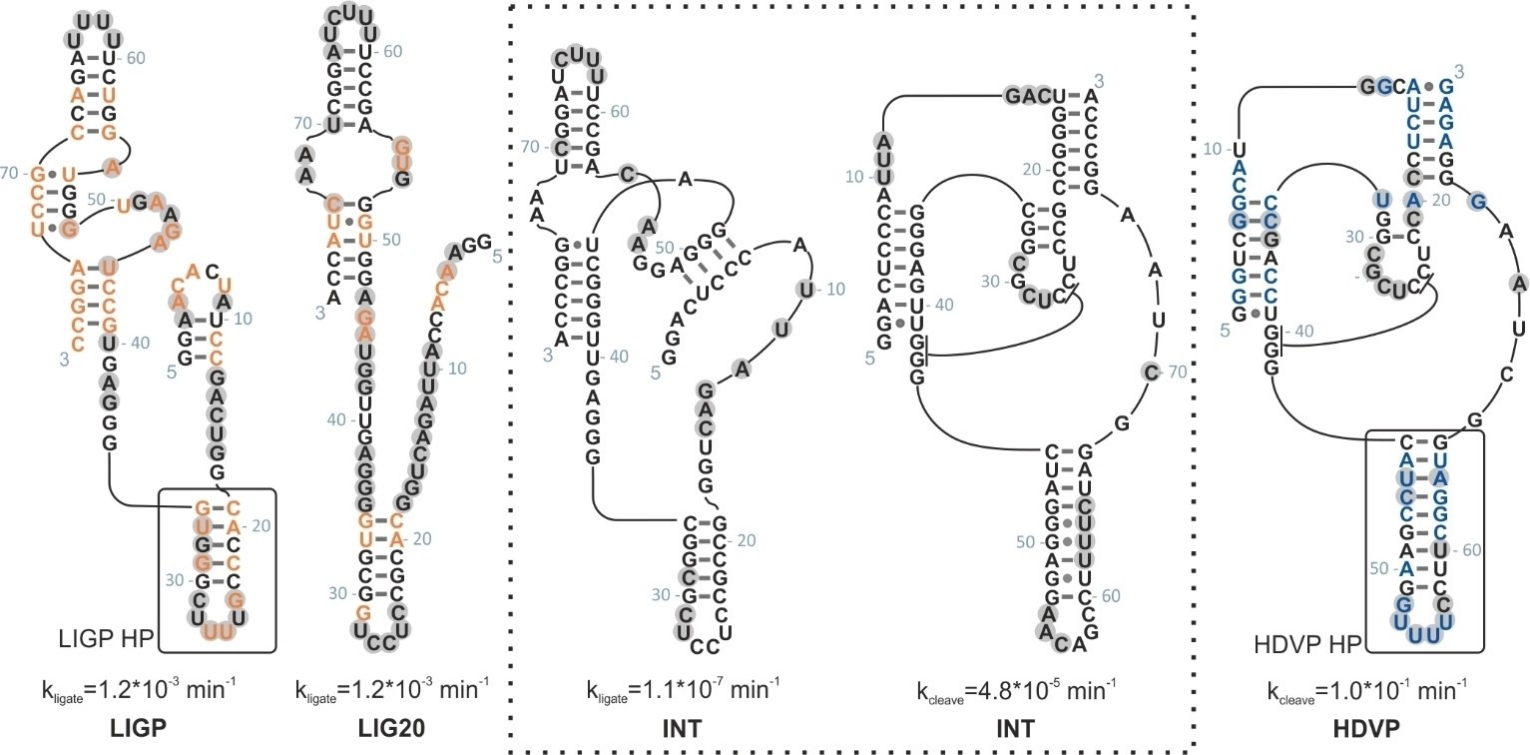
Sequences and predicted secondary structures are shown for LIGP, LIG20, INT, HDVP. Secondary structures of LIGP and LIG20 are derived from NMR and SHAPE data. Secondary structures of INT and HDVP were proposed by the original paper. Grey highlighted nucleobases featured strong SHAPE activities, indicative for labile structural elements. Orange (ligase) and blue (HDV) nucleotides indicate mutations with respect to INT. Secondary structure elements (hair pins, HP) analyzed through NMR imino proton assignment are highlighted with a black box.

To support the NMR analysis, we conducted SHAPE experiments and ten SHAPE patterns are shown in the supporting material (Figure S5). In SHAPE experiments, each detectable band indicates a flexible and therefore unpaired nucleotide. As for some NMR spectra, SHAPE patterns of closely related ribozymes feature similar characteristics. The sequences of LIG1, INT and HDV1 look similar except for the 3’‐end region of HDV1, containing a mutation as well as an overall decrease in intensity for INT. Figure 3 shows mapping of the SHAPE intensities onto theoretically predicted secondary structure models.

SHAPE[^20^, ^21^] (selective 2’‐hydroxyl acylation analyzed by primer extension) profiling of LIGP, LIG20, INT and HDVP are shown in Figure 3. In parts, our new data do not match with the originally proposed secondary structures.[^13^, ^14^, ^15^, ^22^] The new data show co‐population of several conformations, even for the prototype RNAs featuring homogeneous NMR spectra. Thus, we analyzed the secondary structures of LIGP, Lig20, INT and HDVP in greater detail and used 2D NMR spectra to revise the original secondary structures, providing evidence for the existence of a minimum of four different secondary structures along the neutral network.

**Figure 4:**
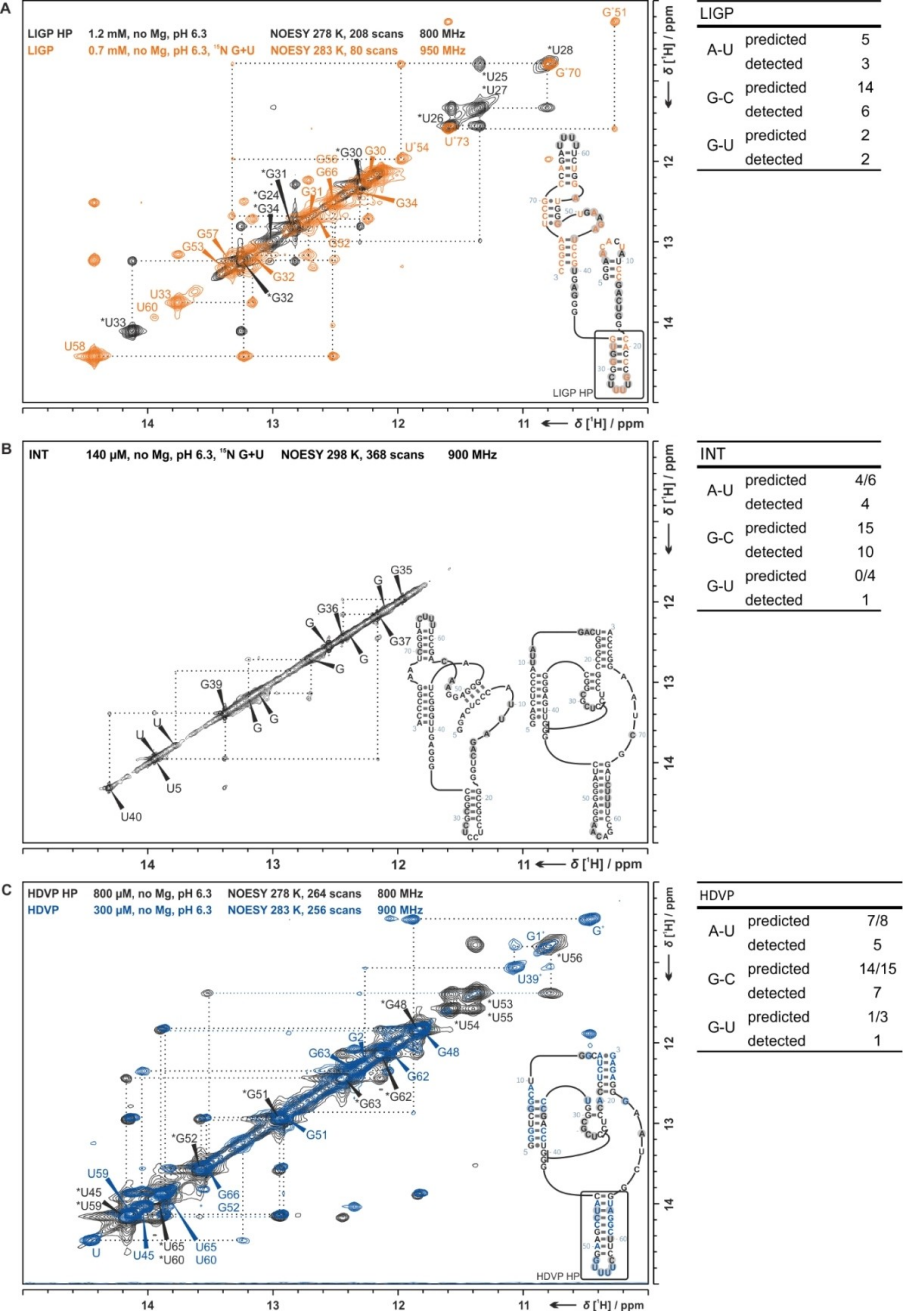
Regions of NMR spectra indicative for imino hydrogen bonds of: A. LIGP (orange) and LIGP HP (black), B. INT, and C. HDVP (blue) and HDVP HP (black) at low temperatures between 278–283 K. Expected (exp) and detected base pairs are listed (slash marks base pairs of different structures presented either here or later on). The samples contained RNA concentrations as indicated (140–1200 μM) with 25 mM potassium phosphate buffer at pH 6.3 with 100 μM NMR reference DSS. Plus signs highlight nucleobase imino protons of non‐canonical base pairs. The 2D ^1^H,^1^H‐NOESY spectra were recorded on Bruker spectrometers with jump‐and‐return solvent suppression, with 80–368 scans and at magnetic fields of 800–950 MHz.

The neutral network contains RNA sequences with about 80 nts in length. Such RNAs are too large for direct detailed NMR analysis of full‐length sequences owing to signal overlap. Therefore, we used a ‘divide‐and‐conquer’ approach for NMR spectra to simplify the signal assignment. In Figure 4, 2D ^1^H,^1^H‐NOESY spectra of the flanking prototype sequences LIGP and HDVP as well as the spectrum of INT are illustrated. The spectra of LIGP and HDVP furthermore cover spectra of their individual secondary structure elements for comparison. For additional 2D spectra and assignment procedures see Supporting Information (Figures S9, S10, S13, S20, S28, S29 and S32). In order to implement the divide‐and‐conquer approach, we designed hairpin models of the RNA sequences LIGP and HDVP. NMR spectra of these hairpin models can be assigned with high confidence, allowing transfer of these initial assignment spectra to the full‐length sequences. This procedure exploits the observation that chemical shifts observed in smaller structural elements remain largely unchanged in larger constructs. We thus investigated unlabeled model hairpins LIGP HP (5’‐CACCCGUU UUCGGGUG‐3’) and HDVP HP (5’‐CAUCCGAAGGUU UUCCUUCGGAUG‐3’). Figure 4A shows the comparison of LIGP and its hairpin. Two dimensional spectra were recorded at 25 °C and 10 °C (see Supporting Information, Figures S9 and S10). Through comparison of the RNA spectra, we assigned LIGP in parts and observed additional signals indicating additional base pair interactions. Even if an unambiguous resonance assignment cannot be achieved, we can derive that these signals belong to the inner part of LIGP. Four signals, two uridines (U) and two guanosines (G), are forming G‐U base pairs due to their characteristic chemical shift around 10–12 ppm. Hence, five Gs and two Us are missing or could not be assigned. We included an additional data point, where the mismatches between two ^1^H NMR spectra are merged into one new spectrum (see Supporting Information, Figure S13). The comparison of LIGP and LIG36 lead to new information about previously unassigned base pairs. Thus, we assigned the stem between G51*U73 and U54*G70. Using these data as restraints for secondary structure prediction by online tools (mFold,[^23^, ^24^, ^25^] RNA structure[^26^, ^27^]), we thus propose a new secondary structure for LIGP, different from the initial suggestion.

The secondary structure of HDVP was likewise analyzed by NMR experiments at room temperature. Additional data recorded at 278–283 K are shown in the Supporting Information (Figures S28 and S29). In addition, we analyzed the structural hairpin HP C43‐G66 as part of the full‐length HDVP. The NMR chemical shift assignment of this hairpin structure helped for the assignment of the HDVP spectra and led to the finding that most signals from hetero‐ and homonuclear experiments of the full‐length construct belong to the C34‐G66 hairpin (Figure 4C). Two of the unassigned signals belong to a G*U base‐pair at the 5’ end of full length HDVP ribozyme forming the catalytic core. Assuming the signals refer to G1 and U39, G2 can be assigned leaving three canonical Gs, two canonical Us and one non‐canonical G unassigned when referring to the originally proposed secondary structure. Furthermore, 1D ^1^H temperature rows of HDVP and HP (see Supporting Information, Figures S27 and S30, S34 and S35) mostly showed higher signal‐to‐noise for imino proton signals at higher temperatures, with few exceptions, e. g. G48 for HDVP and G24 for HP. Especially hairpin imino protons appear to dominate the signal pattern with strong and sharp signals whereas others are less intense and decrease fast at low temperatures. This observation as well as the assignment indicate a highly stable hairpin element in the HDVP secondary structure and a less stable remaining structure.

The 2D spectrum of INT is shown in Figure 4B. As annotated, we assigned strong new signals to base pairs between U40*A3 and G35*C8. This ribozyme was reported to adopt two different structures, each one possessing one catalytic function. The NMR data reveal a higher population of the HDV secondary conformation compared to conformations presumably linked to ligase catalytic activity. In addition, native PAGE experiments show three different folds with one dominating secondary structure. Taken together, these findings suggest the presence of a major populated secondary structure state together with minor populated states.

**Figure 5:**
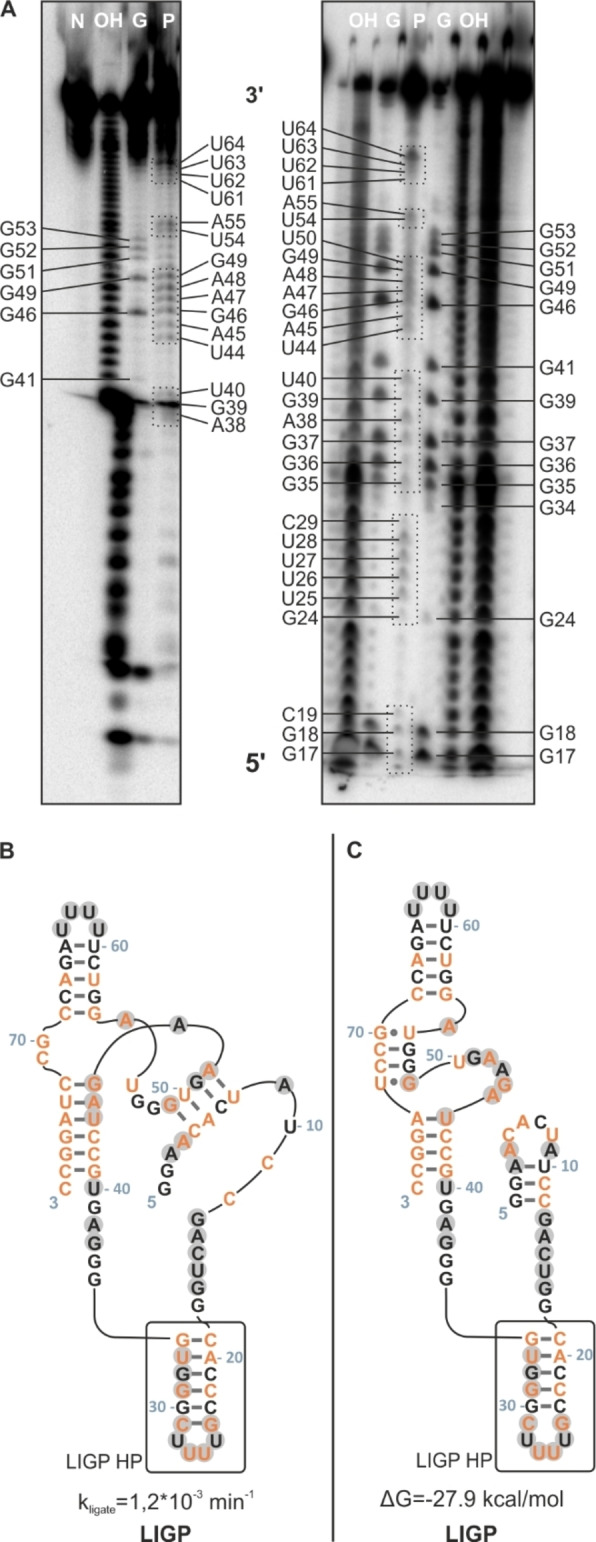
A. In‐line probing data of LIGP. Not all parts of the RNA are displayed in the gel pictures. Native RNA (N), alkaline hydrolysis ladder (OH), RNase T1 ladder (G) and probed RNA (P) are displayed. The denaturing gels with 10 % PAA were casted at 46 W. Bands were imaged via ^32^P‐ATP. B. Originally proposed secondary structure (and sequence) of LIGP. C Revised secondary structure (and sequence) of LIGP as proposed by online tools. Grey highlighted nucleobases featured strong SHAPE activities. Orange nucleobases point out the mutations with respect to INT.

Figure 5 shows the two possible secondary structures for LIGP ribozyme and additional in‐line probing data, collected for this ribozyme. The left structure (B) was proposed by the Bartel group, based on structure predictions from in‐vitro selection, evolution of ligase ribozyme variants (analysis of covariation) and probing experiments. Grey highlighted nucleotides refer to strong SHAPE activities from our experiments. Apparently, some paired nucleobases including U44, G49 and G51 feature SHAPE activity, contradicting previous secondary structure predictions. G31, G32 and U33 show only small activity in SHAPE experiments but as specified above, NMR experiments confirm the existence of the respective hairpin structure. However, with the help of NMR and prediction programs we can revise the secondary structure of LIGP. The second structure (C) is more consistent with the SHAPE and in‐line‐probing data. NMR data provide further evidence for two G*U base pairs, which are to be found in the upper region of the new structure (see G51*U73, U54*G70) whereas the original one lacks G*U base pairs. In addition, a native polyacrylamide gel (see Supporting Information Figure S4) indicates the existence of one dominating secondary structure for the LIGP sequence and a second, less populated fold.

**Figure 6:**
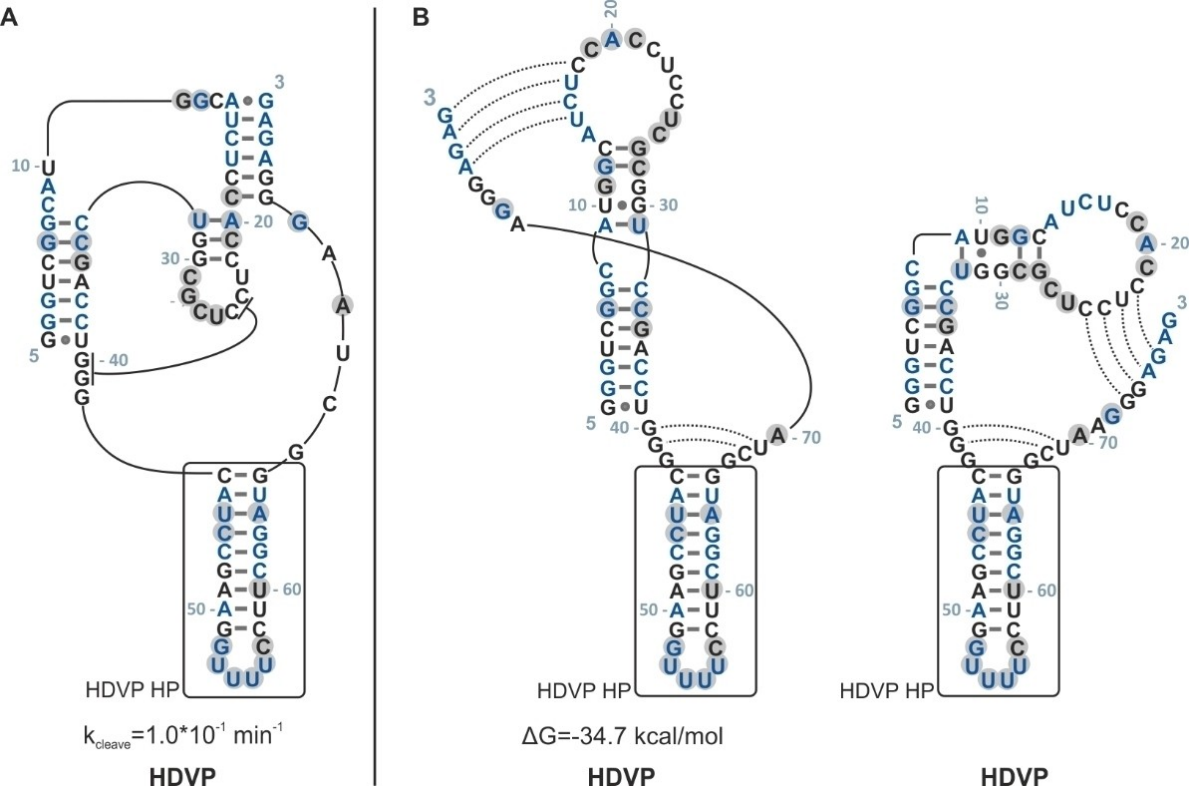
Original in comparison with revised secondary structures (and sequence) of HDVP as proposed by online tools is shown. Grey highlighted nucleobases featured strong SHAPE activities. Blue nucleobases point out the mutations with respect to INT.

NMR and SHAPE data mostly align with the originally proposed structure for the HDVP sequence. Thus, the hairpin HDVP HP C43‐G66 is a stable structural element, which will be present in closely related sequences. The remaining part of this RNA seems to be more flexible besides the catalytically active part, the 5’ end (see Figure 6). This statement is supported by low SHAPE intensities for this RNA apart from the hairpin SHAPE intensities (see Supporting Information, Figure S5). In Figure 6, we suggest two other possible secondary structures for HDVP (B), which reconcile these findings. Migration behavior detected by native polyacrylamide gel further indicates the existence of three different structures, one dominating fold and two equally minor populated folds.

**Figure 7:**
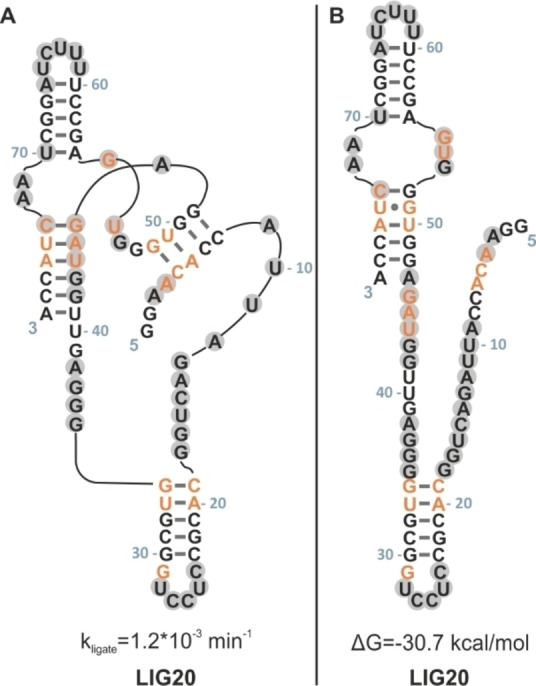
A. Original secondary structure (and sequence) of LIG20. B. Revised secondary structure of LIG20 as proposed by online tools. Grey highlighted nucleobases featured strong SHAPE activities. Orange nucleobases point out the mutations with respect to INT.

Intrinsic changes in the fingerprint region of 1D NMR spectra of LIG22 and LIG20 indicated a major diversification of secondary structure relative to LIGP and LIG36. Due to stronger signals in the LIG20 1D proton spectrum as compared with LIG22, we analyzed this ribozyme by use of 2D NMR experiments. Figure S7 shows 1D and 2D homo‐ and heteronuclear experiments. The findings point to the existence of another secondary structure unlike the original one. Moreover the evidence for the two hairpins C19‐G34 and A56‐U70 indicate, that they are conserved throughout the ligase part of the neutral network, but other parts vary in their arrangement. Figure 7 illustrates original and new suggested secondary structures for LIG20.

## Conclusion

This study tries to delineate the structural impact of stepwise mutations within a neutral network that connects two ribozymes with distinctly different functions as model for the continuous evolution within a potential RNA world. This network was originally devised by Schultes and Bartel and comprises 89 different RNA sequences. In order to reach a comprehensive coverage of RNA structure space to conduct a structure‐function‐analysis, we apply here NMR spectroscopic analysis of 12 sequences and foot‐printing experiments of nine sequences. 20 years after the initial report, we can refine the originally proposed structures. Firstly, we analyzed the secondary structures of particular ribozyme sequences from the neutral network through SHAPE experiments to gain insight into unfolded regions of the RNAs. A partial alignment of original versus SHAPE data indicated unstable secondary structures for the RNAs. Subsequent CD experiments revealed melting points around 60 °C indicative for stable RNA structures. Then, we used NMR experiments focusing on the analysis of imino signals that report on folded, base‐paired nucleotides in the RNA structures. The assignment was simplified by applying the divide‐and‐conquer method, where we first analyzed and assigned the hairpin spectra of LIGP HP (C19‐G34 in full length RNA) and HDVP HP (C34‐G66 in full length RNA) and then used the data to assign the NMR spectra of full length RNAs. The assignment of full length HDVP spectra featured only its HP signals; other signals could not be assigned to the remaining structural elements with certainty. LIGP on the other hand presented signals within the NMR experiments, which covered LIGP HP (C19‐G34) and another hairpin element, G56‐C69. We made use of these data and found a new and slightly different secondary structure for LIGP. This finding was confirmed through the analysis of LIG20. The respective data revealed two hairpin structures, C19‐G34 and A56‐U70, indicating that these structural elements are conserved despite the mutations in sequence. Our analysis indicated that not only the destabilization of HDV structure by mutation (A4) increases ligase activity through increased fold population but also the hairpin structures are stabilized by the following mutations (G29, G46, C73) causing a strong activity increase, too.

Our study applies novel methodology to study even large RNA sequence space in considerable detail. Application of complementary technology sensitive to presence (NMR) or absence (in‐line probing) of base‐pairing at nucleotide resolution is essential to reach a structure‐function relationship. The thus established structure‐function relationship shows that enzymatic function of the ribozymes has to be linked to lowly‐populated conformations since the revised secondary structures do not adopt structural characteristics required for catalytic activity;[^14^, ^28^] as suggested by the original publication through covariation analysis. Our findings might suggest that a system evolving from a prebiotic RNA‐world to the present world might require a fuzzier concept regarding the sequence‐structure‐function correlation than assumed before. Co‐existence of different RNA sequences adopting different conformations and featuring simultaneous functions following the quasi‐species concept may have played an important role when it comes to the survival of the fittest. The ensemble characteristics populating several conformations of a given sequences allows for branching development of life.

## Experimental Section

**Documentation**: For analytical images see Figures S1‐5, S14 and S38 in the Supporting Information.

**RNA sample preparation**: RNAs were prepared by in‐vitro transcription and solid phase synthesis. The latter ones were purchased from Dharmacon™. The RNAs were deprotected, as described on their website. For in‐vitro transcription[^29^, ^30^, ^31^, ^32^] DNA amplified by PCR was used. The DNA templates and primers were purchased at Eurofins Genomics GmbH. PCR yielded 60–90 % product whereas in‐vitro transcriptions yielded 40–60 % product. After transcription with T7 RNA polymerase the RNAs were either purified through preparative PAGE or buffer exchange.^[33]^ The buffer was exchanged for 25 mM NMR potassium phosphate buffer (K_2_HPO_4_/KH_2_PO_4_; pH 6.3) by using centrifugal concentrators with 5k MWCO from Sartorius, if RNA was used for NMR experiments. The RNAs were then folded at 95 °C and 10 % D2O and 100 μM DSS were added. We used Shigemi NMR tubes (Shigemi Inc.). For further information about NMR sample preparation see Schnieders et al.^[16]^

**SHAPE experiments**: RNAs for SHAPE experiments were purified by use of magnetic beads. RNAs were chemically modified with 1M7 at room temperature for 15 min and quenched with Na‐MES (pH 6.0). A reaction mixture of 20 μL contained 10 mM MgCl_2_, 0.06 μM RNA, 50 mM Na‐HEPES (pH 8) and 5 μL 1M7 mixture (4.2 mg/mL; DMSO). The modified RNA was reverse transcribed with Superscript III reverse transcriptase from Invitrogen. For detection of cDNA fragments an ABI 3100 capillary sequencer was used. The data analysis was performed with the HiTRACE^[34]^ MATLAB® toolkit with MATLAB R2017a. Purification and probing experiments were performed as described by Cordero et al.^[35]^

**CD spectroscopic measurements**: Concentrations of RNA samples were adjusted to 100 μM RNA with 25 mM potassium phosphate buffer (NMR samples were used). The measured temperature range was 5 °C–95 °C. CD intensities were measured with a JASCO J‐810 spectropolarimeter. The data was visualized via SigmaPlot™ 12.5 and is shown in the Supporting Information, Figures S12, S22 and S29 in the Supporting Information.

**In‐line probing**: RNAs were in‐vitro transcribed. Leftover free rNTPs were removed by a NAP‐5 column from Merck. The RNAs were precipitated from EtOH with NaOAc and the 5’ ends were dephosphorylated with Shrimp Alkaline Phosphatase (rSAP) from New England Biolabs® (NEB) for 4 h at 37 °C. The 5’ ends were phosphorylated with ^32^P‐ATP by using T4 Polynucleotide Kinase from NEB® for 40 min at 37 °C. The RNAs were then purified via preparative PAGE. Probing was performed with probing buffer (25 mM TRIS, 10 mM MgCl_2_, 50 mM KCl) at room temperature for 15 h. The result was analyzed by denaturing polyacrylamide gels. For imaging phosphor imaging plates and a Typhoon™ Scanner 9400 from GE Healthcare were used.

**NMR spectroscopy experiments**: NMR experiments were performed on Bruker spectrometers with fields ranging from 600 MHz to 950 MHz and from 278 K to 298 K. We applied jump‐return echo experiments from Sklenar and Bax^[19]^ for 1D ^1^H and 2D ^1^H,^1^H‐NOESY NMR spectra. For heteronuclear experiments we used BEST‐TROSY[^36^, ^37^] and SFHMQC^[38]^ pulse sequences. For ^15^N‐labeled samples, decoupling was obtained through a method by Shaka et al.^[39]^ For processing and assignment TopSpin® 4.0.6 was used. For the evaluation of imino proton integrals Simplex Numerica64 was used. All NMR spectra are shown in the Supporting Information, Figures S6–S10, S13, S15, S17–S20, S23–S28, S30–S34, S37 and S39.

## Conflict of interest

The authors declare no conflict of interest.

1

## Supporting information

As a service to our authors and readers, this journal provides supporting information supplied by the authors. Such materials are peer reviewed and may be re‐organized for online delivery, but are not copy‐edited or typeset. Technical support issues arising from supporting information (other than missing files) should be addressed to the authors.

Supporting Information

## Data Availability

The data that support the findings of this study are available from the corresponding author upon reasonable request.
