# Supplementary material for: Mapping the Conformational Landscape of the Neutral Network of RNA Sequences That Connect Two Functional Distinctly Different Ribozymes
Source: Chembiochem. 2022 Feb 15;23(7):e202200022. doi: 10.1002/cbic.202200022 (PMC9305247; doi:10.1002/cbic.202200022)
Supplement: Supplementary file 1 — Supporting Information [file CBIC-23-0-s001.pdf]

ChemBioChem

Supporting Information

Mapping the Conformational Landscape of the Neutral Network of RNA Sequences That Connect Two Functional Distinctly Different Ribozymes

Bozana Knezic, Sara Keyhani-Goldau, and Harald Schwalbe*

Table of Contents

Experimental section 1
LIGP 5
LIG20 13
LIG16 & LIG14 17
LIG1, INT & HDV1 18
HDVP 21

Experimental section

Table S1 DNA sequences of PCR templates and respective primer sequences are listed for LIGP, LIG36, LIG30, LIG18, LIG8, LIG1, INT HDV1, HDV9, HDV17, HDV32 and HDVP. The RNA sequence for each construct is shown below. Primer 1 stands for the forward primer and Primer 2 stands for the reverse primer required for PCR. All sequences are presented in the direction from 5'- to 3'-end.

**LIGP**	**Primer 1**	**Primer 2**
TAATACGACTCACTATAGGAACACTATCCGACTGGCACCCGTTTTCGGGTGGGGAGTGC<br>CTAGAAGTGGGTAGGTCTTTTTAGACCGCCTAGGCC	TAATACGACTCACT<br>ATAGG	GGCCTAGGCGGT<br>CTA
GGAACACUAUCCGACUGGCACCCGUUUUCGGGUGGGGAGUGCCUAGAAGUGGGUAGGUCUUUUUAGACCGCCUAGGCC		
**LIG36**	**Primer 1**	**Primer 2**
TAATACGACTCACTATAGGAACACCATTAGACTGGCACCCCTTTTGGGGTGGGGAGTGC<br>CTAGAGGTGGGTGGGTCTTTTTAGACCAACTAGGCC	TAATACGACTCACT<br>ATAGG	GGCCTAGTTGGT<br>CTAAA
GGAACACCAUUAGACUGGCACCCCUUUUGGGGUGGGGAGUGCCUAGAGGUGGGUGGGUCUUUUUAGACCAACUAGGCC		
**LIG30**	**Primer 1**	**Primer 2**
TAATACGACTCACTATAGGAACACCATTAGACTGGCACCCCTTTTGGGGTGGGGAGTTC<br>CTAGAGGTGGGTGAGTCTTTTCTAGACTAACTAGGA	TAATACGACTCACT<br>ATAGG	TCCTAGTTAGTCT<br>AGAAAA
GGAACACCAUUAGACUGGCACCCCUUUUGGGGUGGGGAGUUCCUAGAGGUGGGUGAGUCUUUUCUAGACUAACUAGGA		
**LIG18**	**Primer 1**	**Primer 2**
TAATACGACTCACTATAGGAACACCATTAGACTGGCACGCCTCCTGGCGTGGGGAGTTG<br>GTCGAGGTGGGTGAGCCTTTTCTAGGCTAACGACCA	TAATACGACTCACT<br>ATAGG	TGGTCGTTAGCC<br>TAGAA
GGAACACCAUUAGACUGGCACGCCUCCUGGCGUGGGGAGUUGGUCGAGGUGGGUGAGCCUUUUCUAGGCUAACGACCA		
**LIG8**	**Primer 1**	**Primer 2**
TAATACGACTCACTATAGGAATCCCATTAGACTGGGCCGCCTCCTGGCGGCGGGAGTTG<br>GGCGAGGGAGGTGAGCCTTTTCTAGGCTAACGCCCA	TAATACGACTCACT<br>ATAGG	TGGGCGTTAGCC<br>TAGA
GGAAUCCCAUUAGACUGGGCCGCCUCCUGGCGGCGGGAGUUGGGCGAGGGAGGUGAGCCUUUUCUAGGCUAACGCCCA		
**LIG1**	**Primer 1**	**Primer 2**
TAATACGACTCACTATAGGAATCCCATTAGACTGGGCCGCCTCCTCGCGGCGGGAGTTG<br>GGCTAGGGAGGAACAGCCTTTTCTAGGCTAAGGCCCA	TAATACGACTCACT<br>ATAGGAAUC	TGGGCCTTAGCC<br>TAGAAAAG
GGAAUCCCAUUAGACUGGGCCGCCUCCUCGCGGCGGGAGUUGGGCUAGGGAGGAACAGCCUUUUCUAGGCUAAGGCCCA		
**INT**	**Primer 1**	**Primer 2**
TAATACGACTCACTATAGGACTCCCATTAGACTGGGCCGCCTCCTCGCGGCGGGAGTTG<br>GGCTAGGGAGGAACAGCCTTTTCTAGGCTAAGGCCCA	TAATACGACTCACT<br>ATAGGACTC	TGGGCCTTAGCC<br>TAGA
GGACUCCCAUUAGACUGGGCCGCCUCCUCGCGGCGGGAGUUGGGCUAGGGAGGAACAGCCUUUUCUAGGCUAAGGCCCA		
**HDV1**	**Primer 1**	**Primer 2**
TAATACGACTCACTATAGGACTCCCATTAGACTGGGCCGCCTCCTCGCGGCGGGAGTTG<br>GGCTAGGGAGGAACAGCCTTTCCTAGGCTAAGGCCCA	TAATACGACTCACT<br>ATAGGAATC	TGGGCCTTAGCC<br>TAGGAAAG
GGACUCCCAUUAGACUGGGCCGCCUCCUCGCGGCGGGAGUUGGGCUAGGGAGGAACAGCCUUUCCUAGGCUAAGGCCCA		
**HDV9**	**Primer 1**	**Primer 2**
TAATACGACTCACTATAGACTCCGATTAGACTGGTCCGCCTCCTCGCGGCCGGAGTTGG<br>GCTAGGGAGGAACAGCCTTCCCTAGGCTAAGGACCA	TAATACGACTCACT<br>ATAGACTCC	TGGTCCTTAGCC<br>TAGGGAAG
GACUCCGAUUAGACUGGUCCGCCUCCUCGCGGCCGGAGUUGGGCUAGGGAGGAACAGCCUUCCCUAGGCUAAGGACCA		
**HDV17**	**Primer 1**	**Primer 2**
TAATACGACTCACTATAGGCTCGGATTAGACTGGTCCGCCTCCTCGCGGCCCGAGCTGG<br>GCATGGGAGGAACAGCCTTCCCATGGCTAAGGACCA	TAATACGACTCACT<br>ATAGGCTC	TGGTCCTTAGCC<br>ATGGGAA
GGCUCGGAUUAGACUGGUCCGCCUCCUCGCGGCCCGAGCUGGGCAUGGGAGGAACAGCCUUCCCAUGGCUAAGGACCA		

HDV32	Primer 1	Primer 2
TAATACGACTCACTATAGGGTCGGCATGGCTGCTCCACCTCCTCGCGGTCCGACCTGGG CATGGGAAGGTTAGCCTTCCCATGGCTAAGGGAGCA	TAATACGACTCACT ATAGGGTC	TGCTCCCTTAGCC ATGGG
GGGUCGGCAUGGCUGCUCCACCUCCUCGCGGUCCGACCUGGGCAUGGGAAGGUUAGCCUUCCCAUGGCUAAGGGAGCA		
**HDVP**	**Primer 1**	**Primer 2**
TAATACGACTCACTATAGGGTCGGCATGGCATCTCCACCTCCTCGCGGTCCGACCTGGG CATCCGAAGGTTTTCCTTCGGATGGCTAAGGGAGAG	TAATACGACTCACT ATAGG	CTCTCCCTTAGCC ATCCGAA
GGGUCGGCAUGGCAUCUCCACCUCCUCGCGGUCCGACCUGGGCAUCCGAAGGUUUUCCUUCGGAUGGCUAAGGGAGAG		

Table S2 DNA sequences of PCR templates and respective primer sequences are listed for LIG22, LIG20, LIG16, LIG14, LIG12 and LIG10. The RNA sequence for each construct is shown below. Primers 1 and 3 indicate forward primers and Primers 2 and 4 indicate reverse primers required for PCR based on Cordero et al.[1] *All sequences are presented in the direction from 5'- to 3'-end.*

LIG22	Primer 1	Primer 2
TAATACGACTCACTATAGGAACACCATTAGACTGGCACGCCTCCTGG CGTGGGGAGTTGCTAGAGGTGGGTGAGCCTTTTCTAGGCTAACTAGC A	TAATACGACTCACTATAGG AACACCATTAGACTGGCA	CCCCACGCCAGGAGGCGT GCCAGTCTAATGGTGT
	**Primer 3**	**Primer 4**
GGAACACCAUUAGACUGGCACGCCUCCUGGCGUGGGGAGUUGCU AGAGGUGGGUGAGCCUUUUCUAGGCUAACUAGCA	TGGCGTGGGGAGTTGCTA GAGGTGGGTGAGCCTT	TGCTAGTTAGCCTAGAAAA GGCTCACCCACCTCTAGCA A
**LIG20**	**Primer 1**	**Primer 2**
TAATACGACTCACTATAGGAACACCATTAGACTGGCACGCCTCCTGG CGTGGGGAGTTGGTAGAGGTGGGTGAGCCTTTTCTAGGCTAACTACC A	TAATACGACTCACTATAGG AACACCATTAGACTGGCA	CCCCACGCCAGGAGGCGT GCCAGTCTAATGGTGT
	**Primer 3**	**Primer 4**
GGAACACCAUUAGACUGGCACGCCUCCUGGCGUGGGGAGUUGGU AGAGGUGGGUGAGCCUUUUCUAGGCUAACUACCA	TGGCGTGGGGAGTTGGTA GAGGTGGGTGAGCCTT	TGGTAGTTAGCCTAGAAAA GGCTCACCCACCTCTACCA A
**LIG16**	**Primer 1**	**Primer 2**
TAATACGACTCACTATAGGAACACCATTAGACTGGGACGCCTCCTGG CGTCGGGAGTTGGTCGAGGTGGGTGAGCCTTTTCTAGGCTAACGACC A	TAATACGACTCACTATAGG AACACCATTAGACTGGGA C	CCCGACGCCAGGAGGCGT CCCAGTCTAATGGTGT
	**Primer 3**	**Primer 4**
GGAACACCAUUAGACUGGGACGCCUCCUGGCGUCGGGAGUUGGU CGAGGUGGGUGAGCCUUUUCUAGGCUAACGACCA	TGGCGTCGGGAGTTGGTC GAGGTGGGTGAGCCTT	TGGTCGTTAGCCTAGAAAA GGCTCACCCACCTCGACCA A
**LIG14**	**Primer 1**	**Primer 2**
TAATACGACTCACTATAGGAACACCATTAGACTGGGACGCCTCCTGG CGTCGGGAGTTGGGCGAGGTGGGTGAGCCTTTTCTAGGCTAACGCC CA	TAATACGACTCACTATAGG AACACCATTAGACTGGGA C	CCCGACGCCAGGAGGCGT CCCAGTCTAATGGTGT
	**Primer 3**	**Primer 4**
GGAACACCAUUAGACUGGGACGCCUCCUGGCGUCGGGAGUUGGG CGAGGUGGGUGAGCCUUUUCUAGGCUAACGCCCA	TGGCGTCGGGAGTTGGGC GAGGTGGGTGAGCCTT	TGGGCGTTAGCCTAGAAAA GGCTCACCCACCTCGCCCA A
**LIG12**	**Primer 1**	**Primer 2**
TAATACGACTCACTATAGGAACACCATTAGACTGGGCCGCCTCCTGG CGGCGGGAGTTGGGCGAGGTGGGTGAGCCTTTTCTAGGCTAACGCC CA	TAATACGACTCACTATAGG AACACCATTAGACTGGGC C	CCCGCCGCCAGGAGGCGG CCCAGTCTAATGGTGT
	**Primer 3**	**Primer 4**
GGAACACCAUUAGACUGGGCCGCCUCCUGGCGGCGGGAGUUGGG CGAGGUGGGUGAGCCUUUUCUAGGCUAACGCCCA	TGGCGGCGGGAGTTGGG CGAGGTGGGTGAGCCTT	TGGGCGTTAGCCTAGAAAA GGCTCACCCACCTCGCCCA A
**LIG10**	**Primer 1**	**Primer 2**
TAATACGACTCACTATAGGAATACCATTAGACTGGGCCGCCTCCTGG CGGCGGGAGTTGGGCGAGGTAGGTGAGCCTTTTCTAGGCTAACGCC CA	TAATACGACTCACTATAGG AATACCATTAGACTGGGC C	CCCGCCGCCAGGAGGCGG CCCAGTCTAATGGTAT
	**Primer 3**	**Primer 4**
GGAAUACCAUUAGACUGGGCCGCCUCCUGGCGGCGGGAGUUGGG CGAGGUAGGUGAGCCUUUUCUAGGCUAACGCCCA	TGGCGGCGGGAGTTGGG CGAGGTAGGTGAGCCTT	TGGGCGTTAGCCTAGAAAA GGCTCACCTACCTCGCCCA

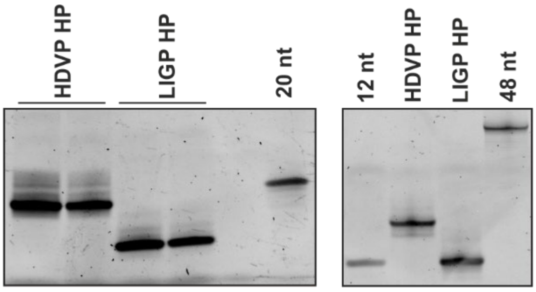

Figure S1 Denaturing 20% polyacrylamide gels with purchased RNAs LIGP HP and HDVP HP are shown. The loaded amount of RNA was 13 pmol. Gels were visualized with GelRed and Bio Rad ChemiDoc XRS+.

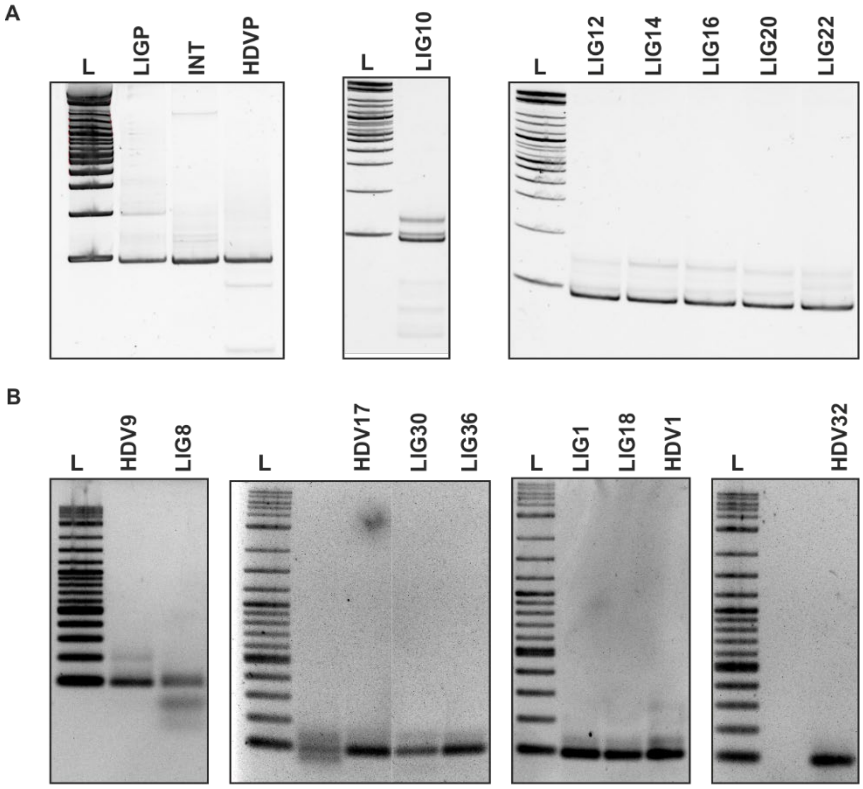

Figure S2 A Native 12% polyacrylamide gel with PCR products of LIGP, INT, HDVP, LIG10, LIG12, LIG14, LIG16, LIG20 and Lig22. Gels were visualized via GelRed and Bio Rad ChemiDoc XRS+. B 1.5 % agarose gels with PCR products of HDV9, LIG8, HDV17, LIG30, LIG36, LIG1, LIG18, HDV1, HDV32. Gels were visualized via GelRed and iNTAS imager.

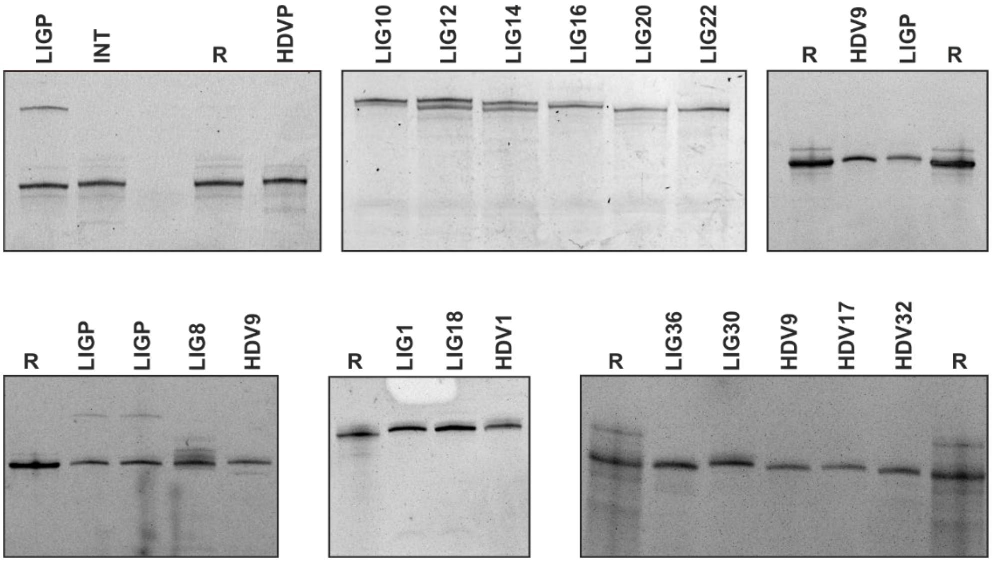

Figure S3 denaturing 15% polyacrylamide gels with purified in-vitro transcription products of LIGP, LIG36, LIG30, LIG22, LIG20, LIG18, LIG16, LIG14, LIG12, LIG10, LIG1, INT, HDV1, HDV9, HDV17, HDV32 and HDVP. LIGP showed a large by-product, which was removed through preparative PAGE (compare second and first row of gel pictures). Gels were visualized via GelRed and Bio Rad ChemiDoc XRS+.

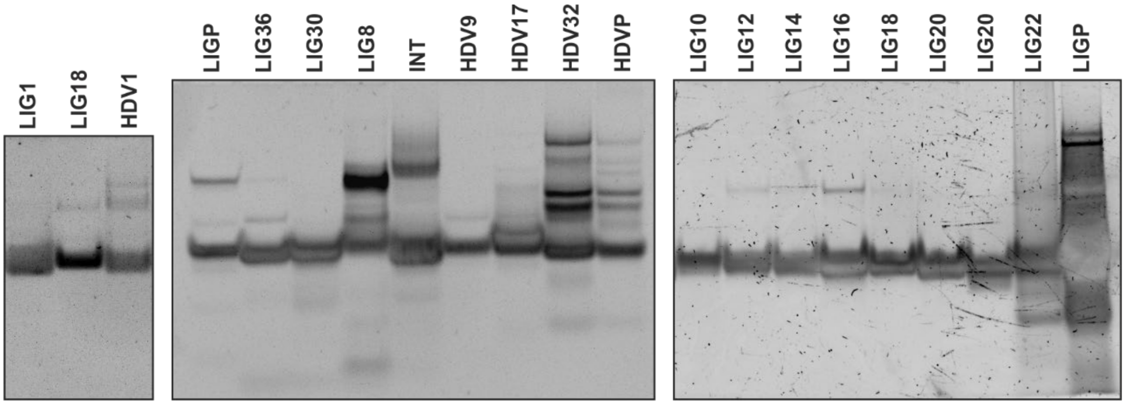

Figure S4 Native 10% polyacrylamide gel with NMR samples of purified and folded in-vitro transcription products of LIGP, LIG36, LIG30, LIG22, LIG20, LIG18, LIG16, LIG14, LIG12, LIG10, LIG8, LIG1, INT, HDV1, HDV9, HDV17, HDV32 and HDVP. All NMR samples display a dominating fold except for LIG8 and INT containing two different structures with comparable populations. Gels were visualized via UV-shadowing.

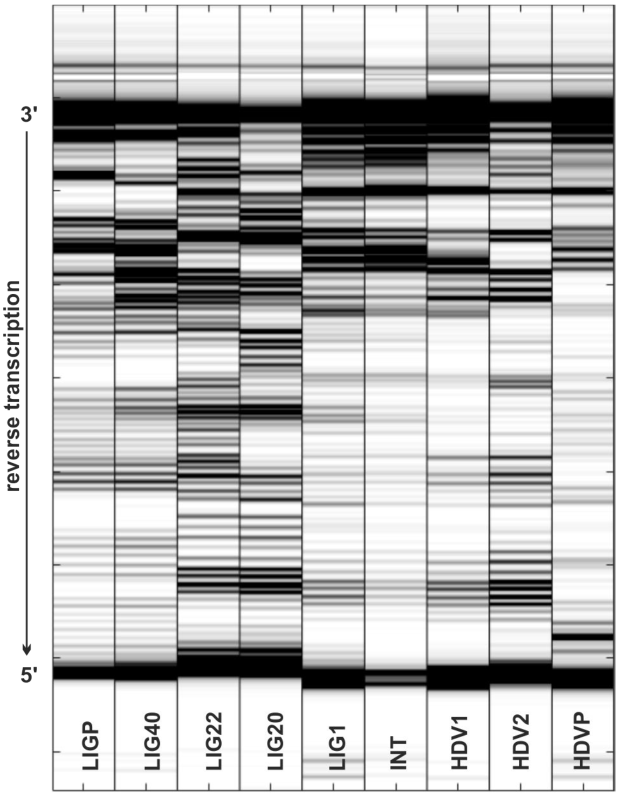

Figure S5 SHAPE patterns of LIGP, LIG40, LIG22, LIG20, LIG1, INT, HDV1, HDV2, HDVP. The RNAs were modified with 1M7, reverse transcribed and the cDNA fragments were analyzed with an ABI 3100 capillary sequencer. The SHAPE patterns were visualized with HiTRACE[2] MATLAB® toolkit with MATLAB R2017a.

LIGP

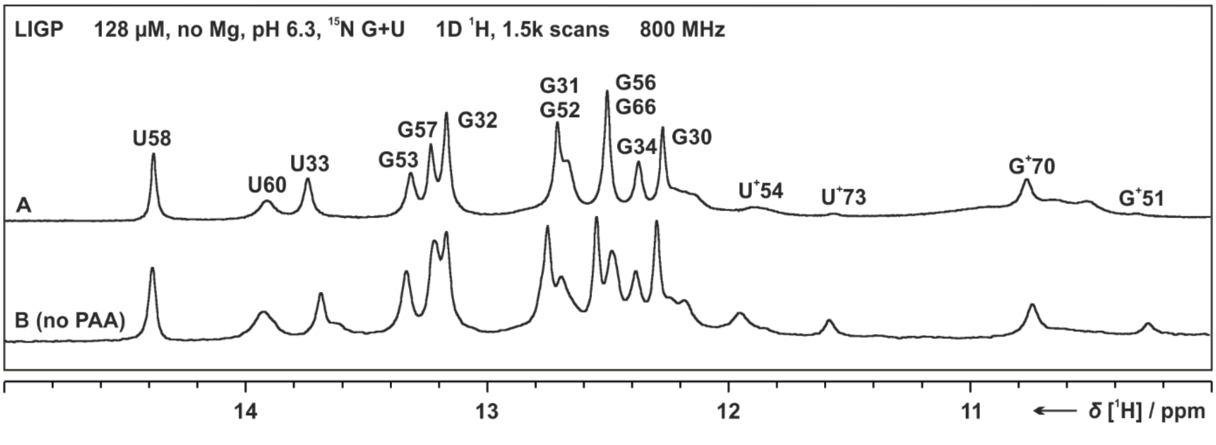

Figure S6 Imino regions of 1D ^{1}H NMR spectra of LIGP. The sample was analyzed first after fast purification with a centrifugal concentrator (B) and it was then purified via preparative PAGE (A). Plus signs highlight nucleobase imino protons of non-canonical base pairs. The spectra were measured with a Bruker spectrometer at 298 K and 900 MHz (A)/800 MHz (B) with 1k (A)/1.5k (B) scans and a spectral width of 24ppm. The samples contained as indicated 128 µM RNA with 25 mM potassium phosphate buffer (pH 6.3), 100 µM NMR reference DSS (A) and 10% D_2O.

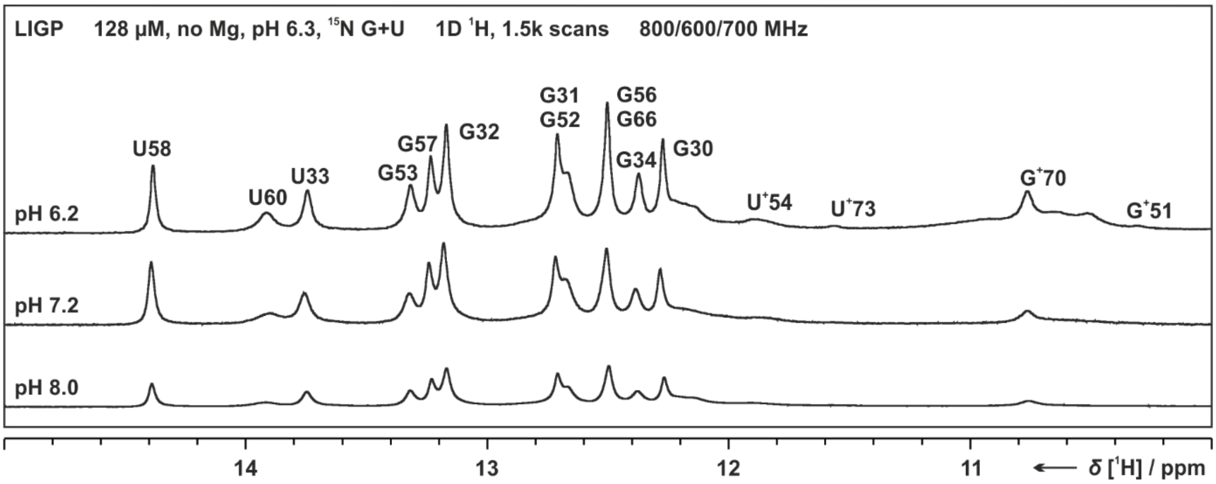

Figure S7 Imino regions 1D ^{1}H NMR spectra of LIGP at different pH levels. The pH levels were set by varying buffer composition of K_2HPO_4/KH_2PO_4 with a concentration of 25 mM. Plus signs highlight nucleobase imino protons of non-canonical base pairs. The spectra were measured with a Bruker spectrometer at 298 K and 800 MHz/600 MHz/700 MHz with 1.5k scans and a spectral width of 24ppm. The samples contained as indicated 128 µM RNA with 25 mM potassium phosphate buffer (pH 6.3) and 10% D_2O.

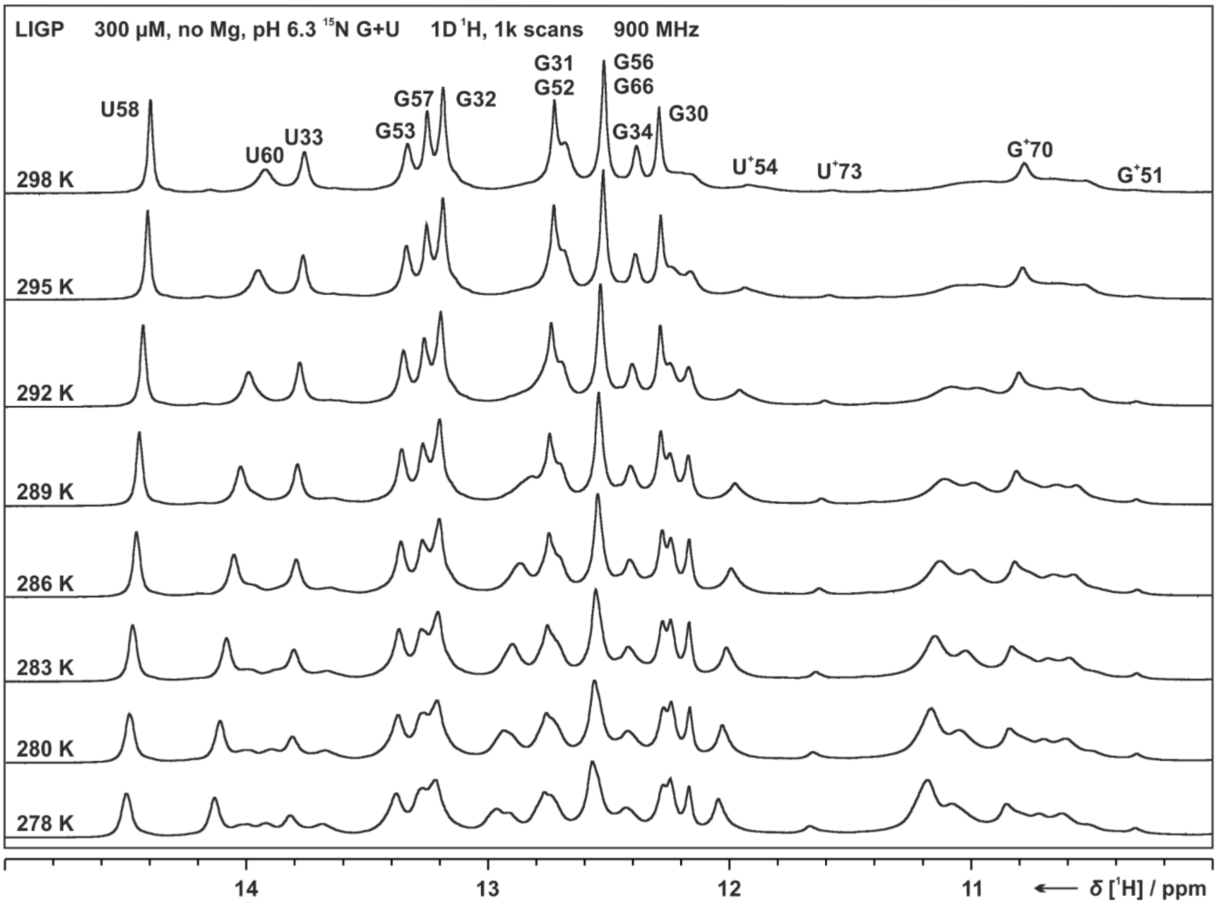

Figure S8 Imino regions 1D ^{1}H NMR spectra of LIGP at different temperatures ranging from 278 K to 298 K. Plus signs highlight nucleobase imino protons of non-canonical base pairs. The spectra were measured with a Bruker spectrometer at 900 MHz with 1k scans and a spectral width of 24ppm. The sample contained as indicated 300 µM RNA with 25 mM potassium phosphate buffer (pH 6.3), 100 µM NMR reference DSS and 10% D_2O.

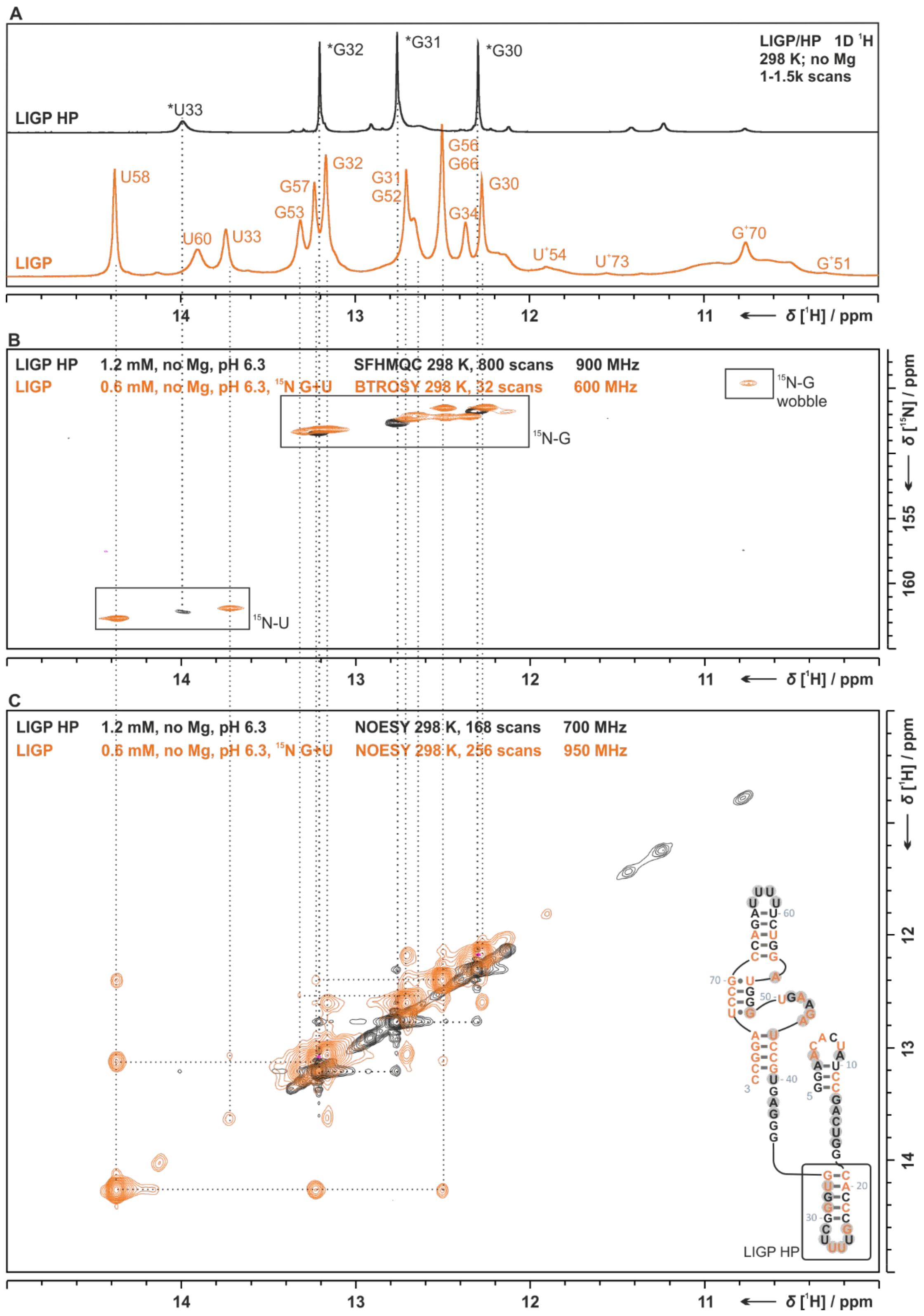

Figure S9 Imino regions of LIGP (orange) and LIGP HP (black) NMR spectra at room temperature (298 K). The samples contained as indicated 1200 µM (LIGP HP) and 600 µM (LigP) RNA with 25 mM potassium phosphate buffer at pH 6.3 with 100 µM NMR reference DSS. Plus signs highlight nucleobase imino protons of non-canonical base pairs. Spectra were recorded on Bruker spectrometers. ***A*** *1D ^{1}H spectrum recorded with 1.5k scans and at a field of 900/950 MHz.* ***B*** *2D $^{1}H,^{15}N$-BEST-TROSY spectrum recorded with 32/800 scans and at a field of 600/900 MHz.* ***C*** *2D $^{1}H,^{1}H$-NOESY spectrum recorded with 256/168 scans and at a field of 950/700 MHz.*

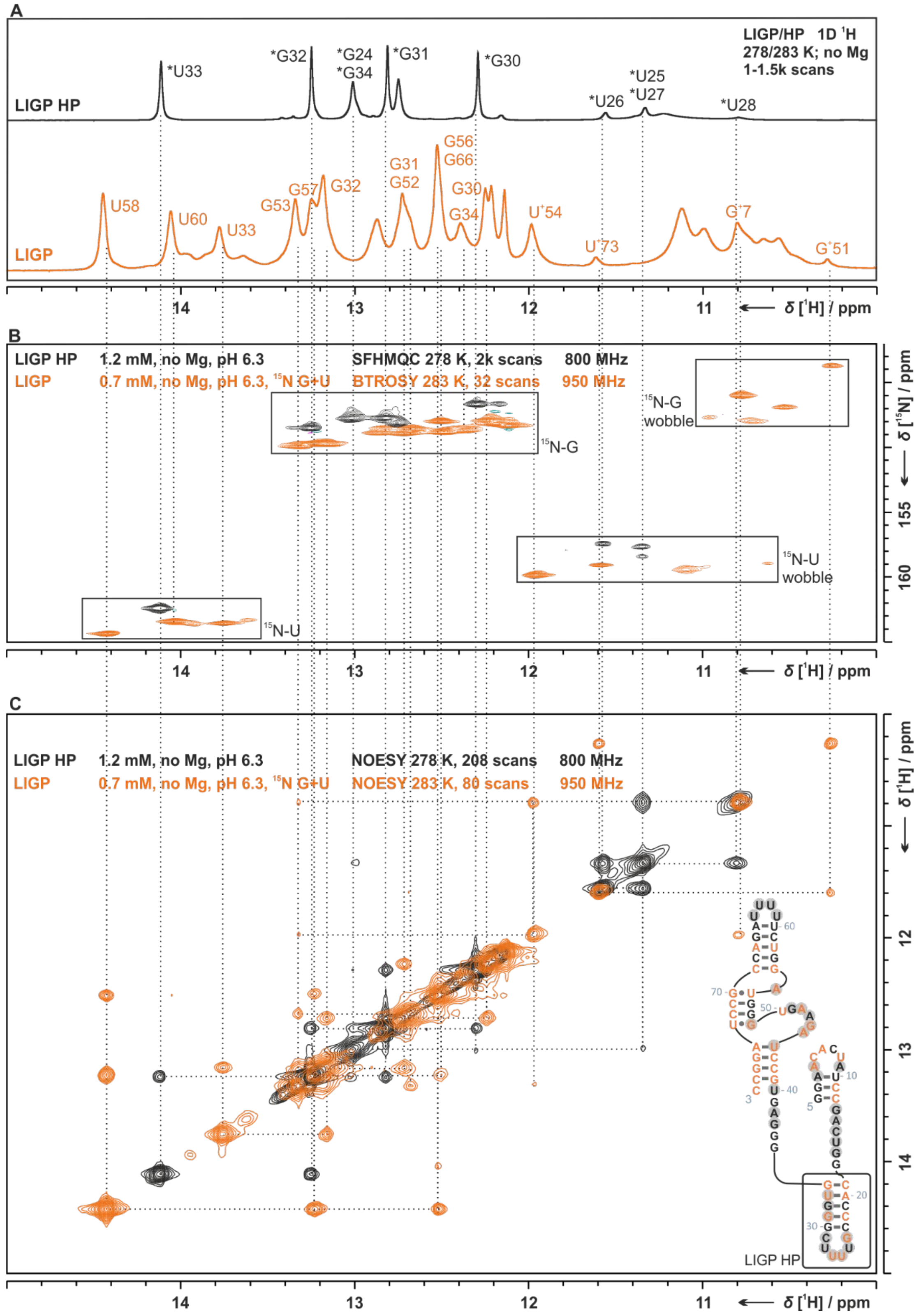

Figure S10 Imino regions of LIGP (orange) and LIGP HP (black) NMR spectra at low temperature. The samples contained as indicated 1200 µM (LIGP HP) and 700 µM (LIGP) RNA with 25 mM potassium phosphate buffer at pH 6.3 with 100 µM NMR reference DSS. Plus signs highlight nucleobase imino protons of non-canonical base pairs. Spectra were recorded on Bruker spectrometers. ***A*** *1D ^{1}H spectrum recorded with 1.5k/1k scans at 278/283 K and 900/950 MHz,* ***B*** *2D $^{1}H,^{15}N$-BEST-TROSY/SFHMQC spectrum recorded with 32/2k scans at 278/283 K and 950/800 MHz.* ***C*** *2D $^{1}H,^{1}H$-NOESY spectrum recorded with 80/208 scans at 278/283 K and 950/800 MHz.*

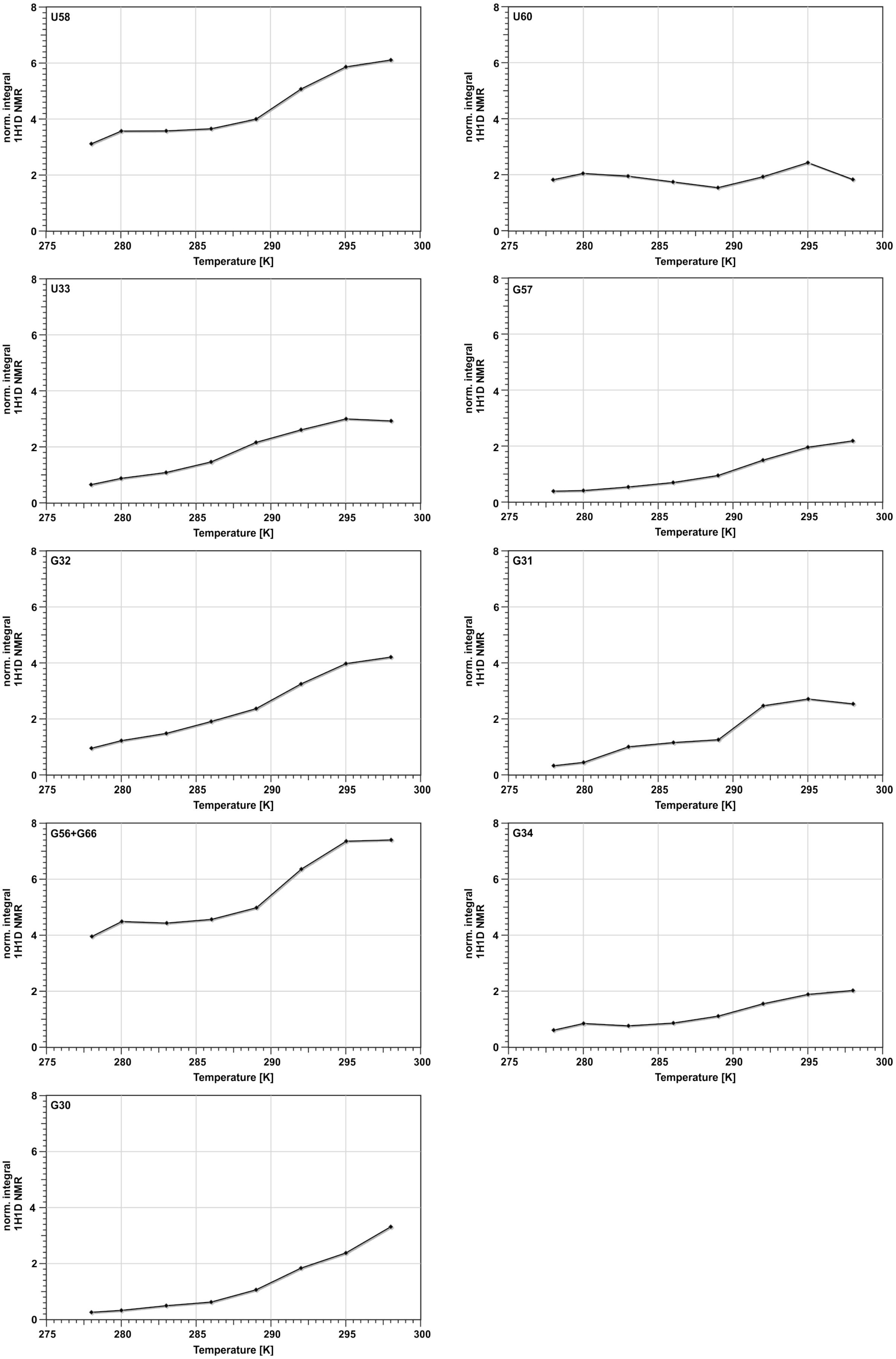

Figure S11 Normalized imino proton signal integral of LIGP over temperature. The integrals were normalized to the DSS proton signal (9 protons) at 0ppm. The temperature range was 278 K-298 K.

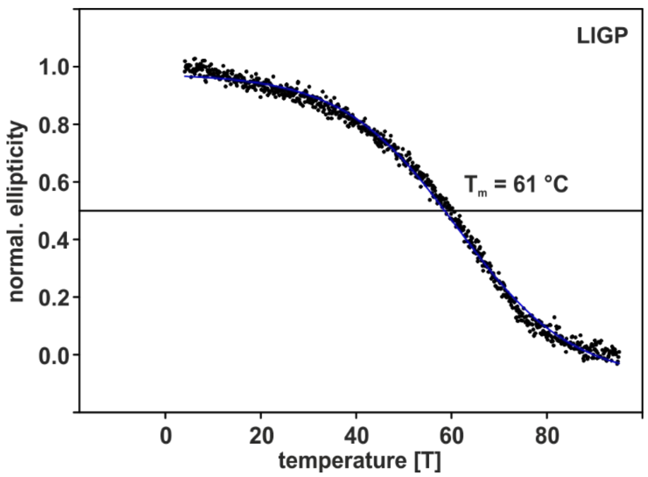

Figure S12 CD melting curve of LIGP was recorded with a JASCO J-810 spectropolarimeter. The sample contained 100 µM NMR sample of the RNA. Molar ellipticity is shown as a function of temperature. T_m was obtained from a sigmoidal fit.

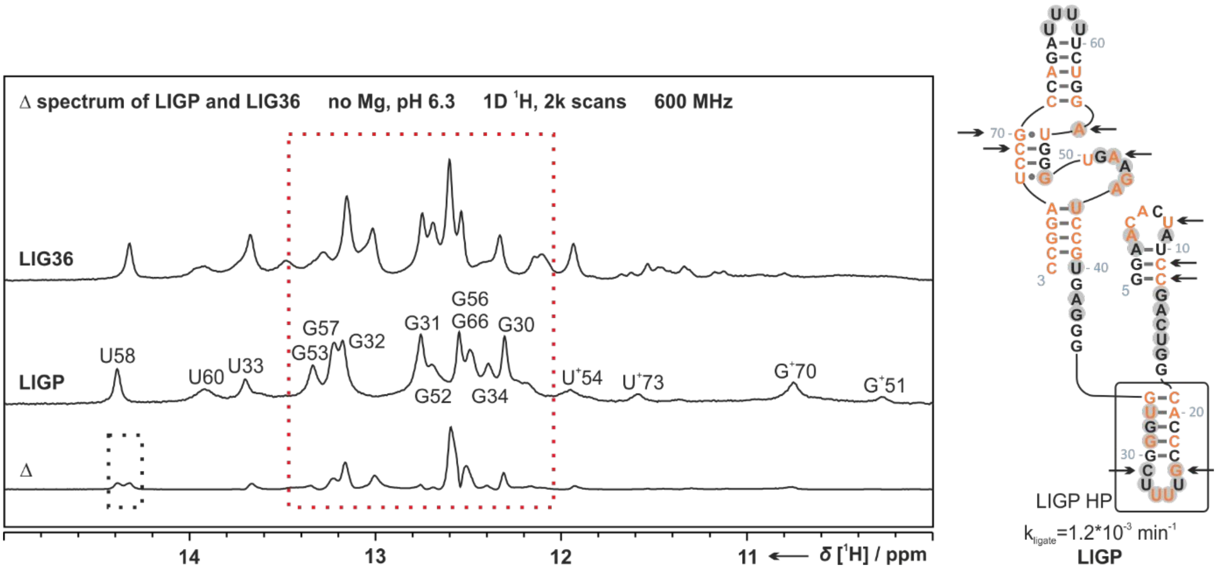

Figure S13 Imino regions 1D ^{1}H NMR spectra of LIGP and LIG36. Plus signs highlight nucleobase imino protons of non-canonical base pairs. The spectra were measured with a Bruker spectrometer at 283 K and 600 MHz with 2k scans and a spectral width of 24ppm. The samples contained 86 µM (LIGP) and 132 µM (LIG36) RNA with 25 mM potassium phosphate buffer (pH 6.3), 100 µM NMR reference DSS and 10% D_2O. The signal intensities were normalized. The difference spectrum (Δ) is shown as a calculated power spectrum. The red dashed box indicates differences in the spectra by comparison. The black dashed box shows an artefact. The 2nd structure of LIGP is shown. Arrows highlight nucleotides different to LIG36.

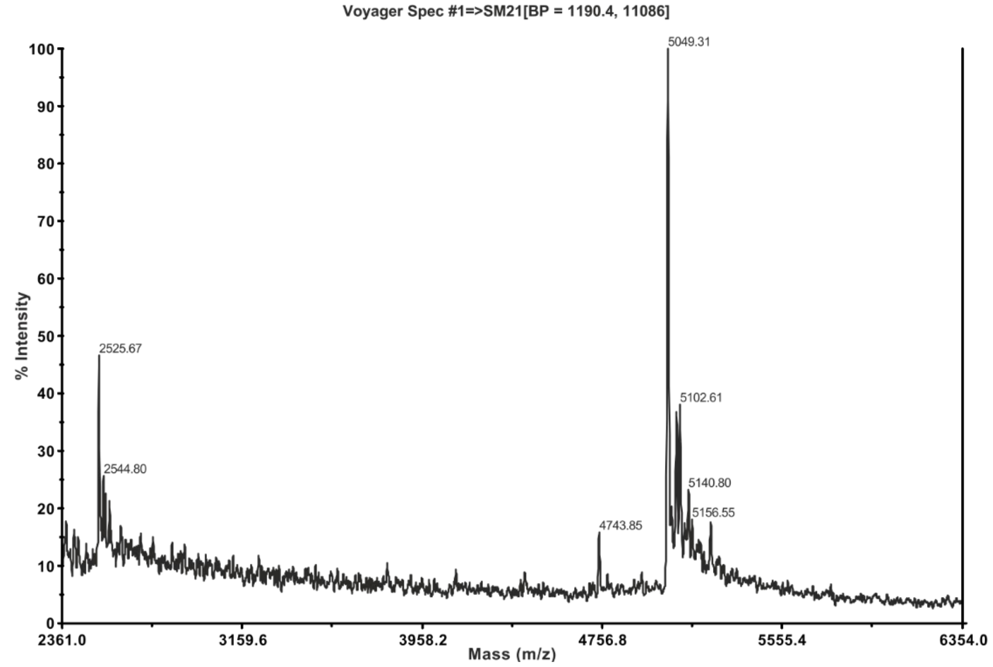

Figure S14 Mass spectrum of LIGP HP. It was recorded through Voyager-DE STR from Applied Biosystems (MALDI). The calculated mass for the 16 nt RNA was 5050.0 u.

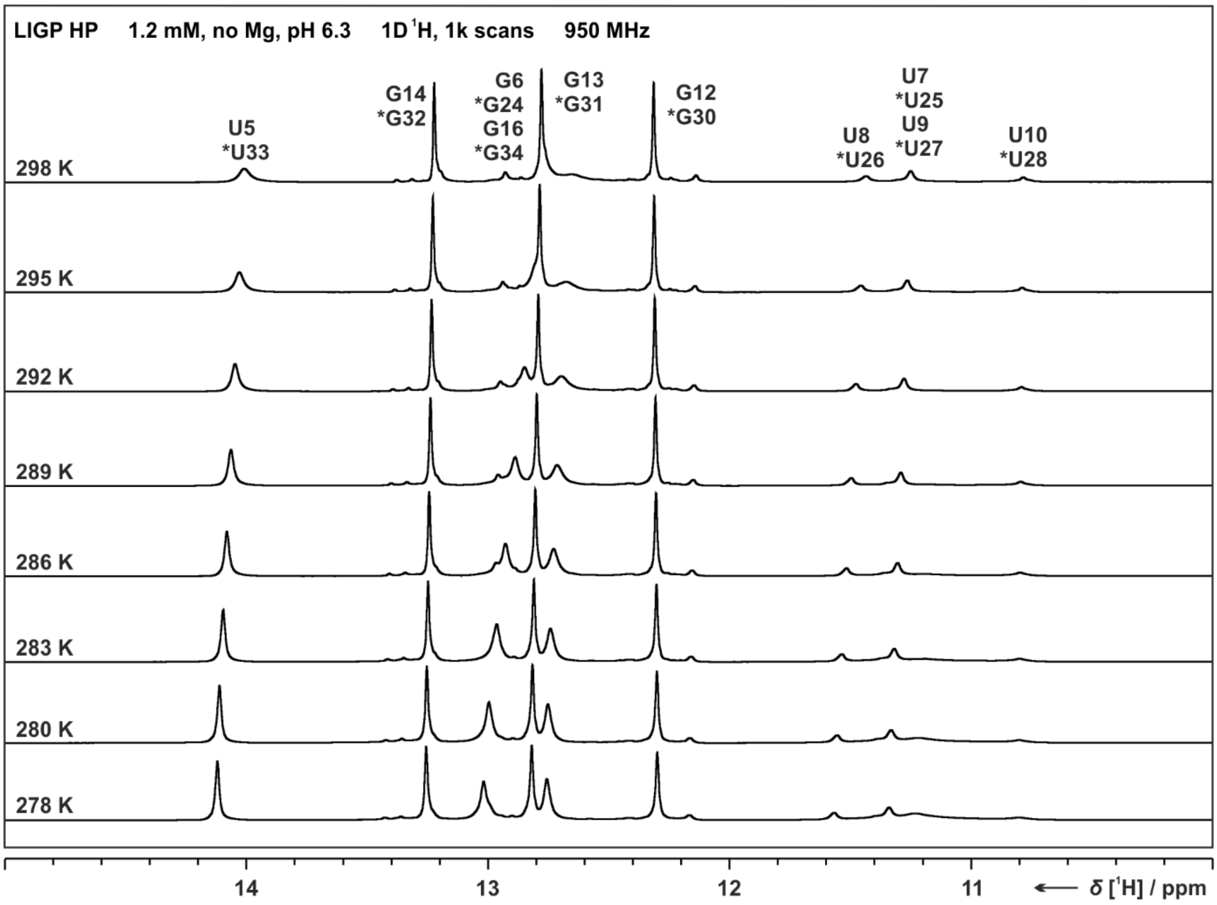

Figure S15 Imino regions 1D ^{1}H NMR spectra of LIGP HP at different temperatures ranging from 278 K to 298 K. Stars indicate nucleotide nomenclature in accordance with the full length construct LIGP. The spectra were measured with a Bruker spectrometer at 950 MHz with 1.5k scans and a spectral width of 24ppm. The sample contained as indicated 1200 µM RNA with 25 mM potassium phosphate buffer (pH6.3), 100 µM DSS and 10% D_2O.

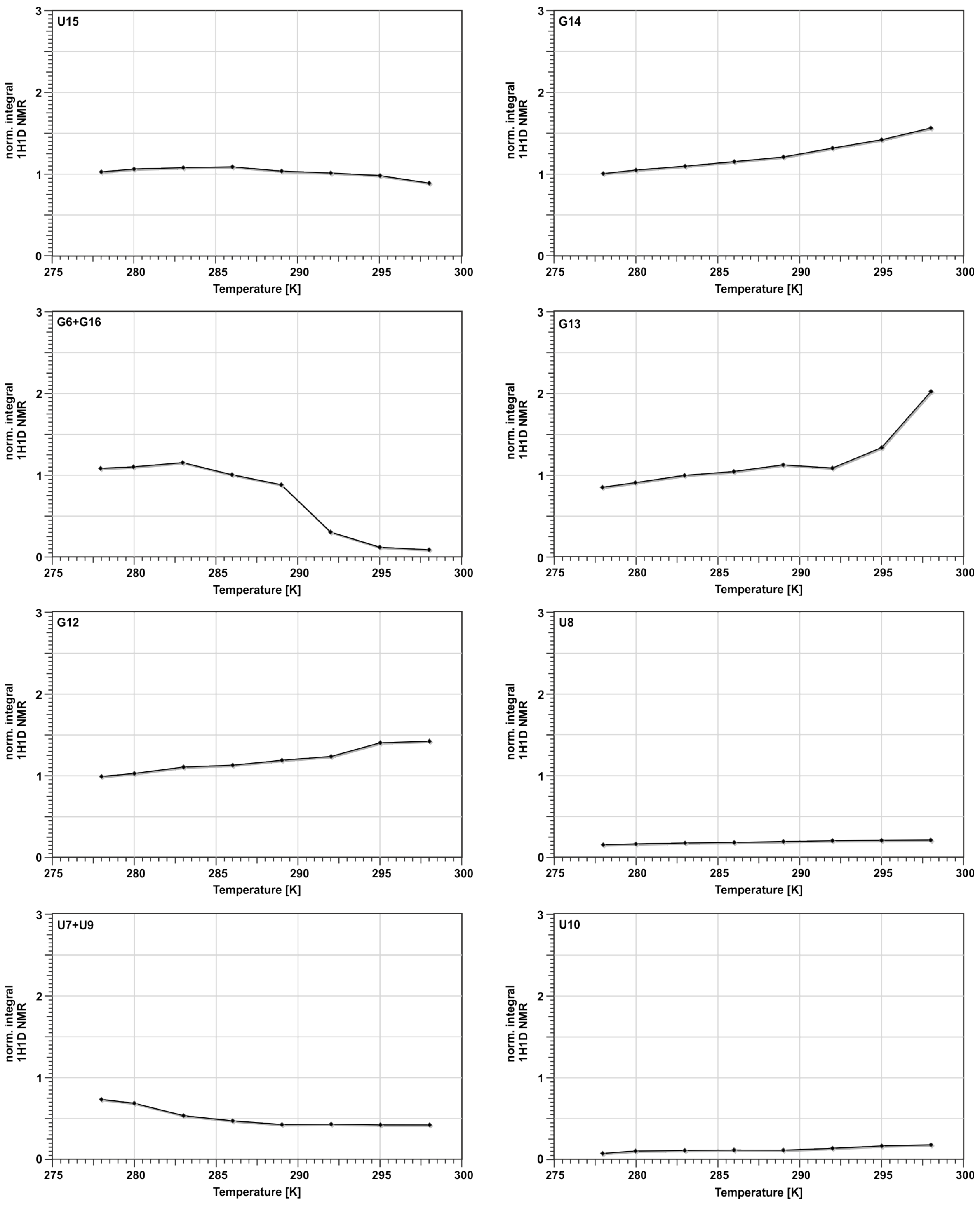

Figure S16 Normalized imino proton signal integral of LIGP HP over temperature. The integrals were normalized to the DSS proton signal (9 protons) at 0ppm. The temperature range was 278 K-298 K.

LIG20

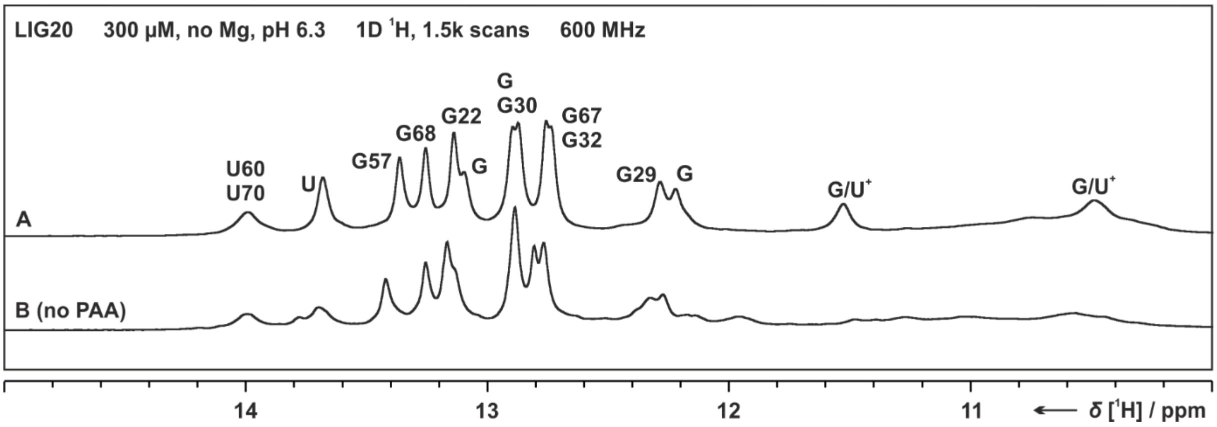

Figure S17 Imino regions 1D ^{1}H NMR spectra of LIG20. The sample was analyzed first after fast purification with a centrifugal concentrator (B) and it was then purified via preparative PAGE (A). Plus signs highlight nucleobase imino protons of non-canonical base pairs. The spectra were measured with a Bruker spectrometer at 298 K and 600 MHz with 1.5k scans and a spectral width of 24ppm. The samples contained as indicated 300 µM RNA with 25 mM potassium phosphate buffer (pH 6.3), 100 µM NMR reference DSS (A) and 10% D_2O.

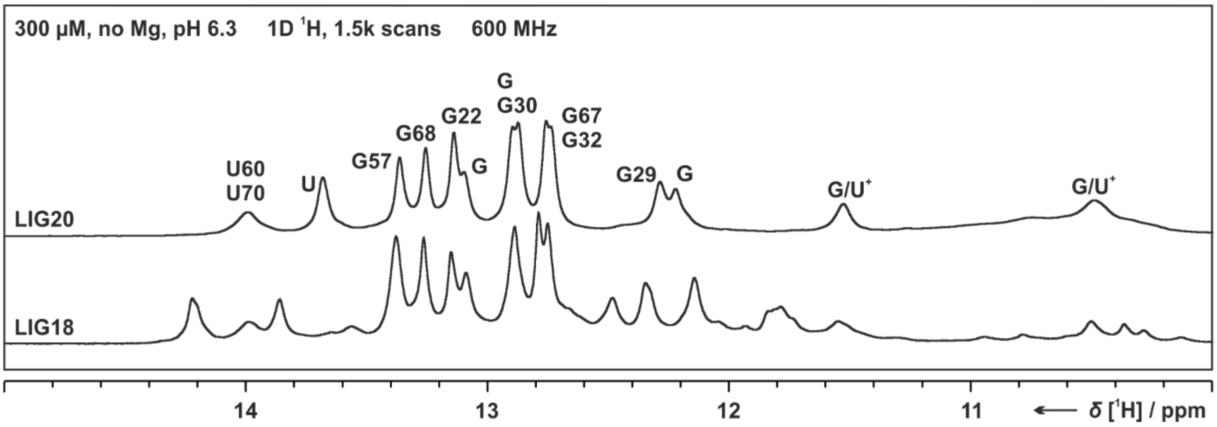

Figure S18 Imino regions 1D ^{1}H NMR spectra of LIG18 and LIG20. Plus signs highlight nucleobase imino protons of non-canonical base pairs. The spectra were measured with a Bruker spectrometer at 298 K and 600 MHz (LIG20)/800 MHz (LIG18) with 1.5k scans and a spectral width of 24ppm. The samples contained as indicated 300 µM RNA with 25 mM potassium phosphate buffer (pH 6.3), 100 µM NMR reference DSS (LIG20) and 10% D_2O.

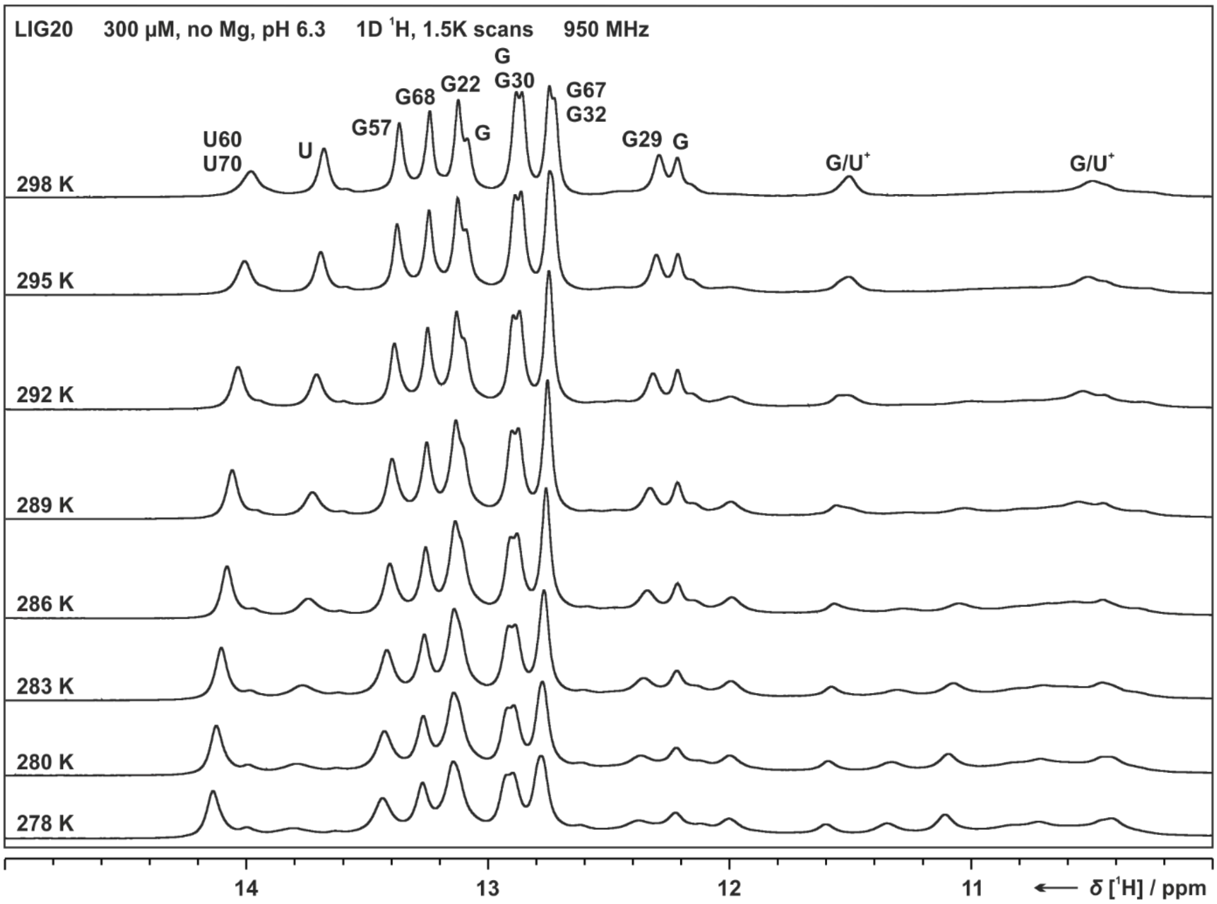

Figure S19 Imino regions 1D ^{1}H NMR spectra of LIG20 at different temperatures ranging from 278 K to 298 K. Plus signs highlight nucleobase imino protons of non-canonical base pairs. The spectra were measured with a Bruker spectrometer at 950 MHz with 1.5k scans and a spectral width of 24ppm. The sample contained as indicated 300 µM RNA with 25 mM potassium phosphate buffer (pH 6.3), 100 µM NMR reference DSS and 10% D_2O.

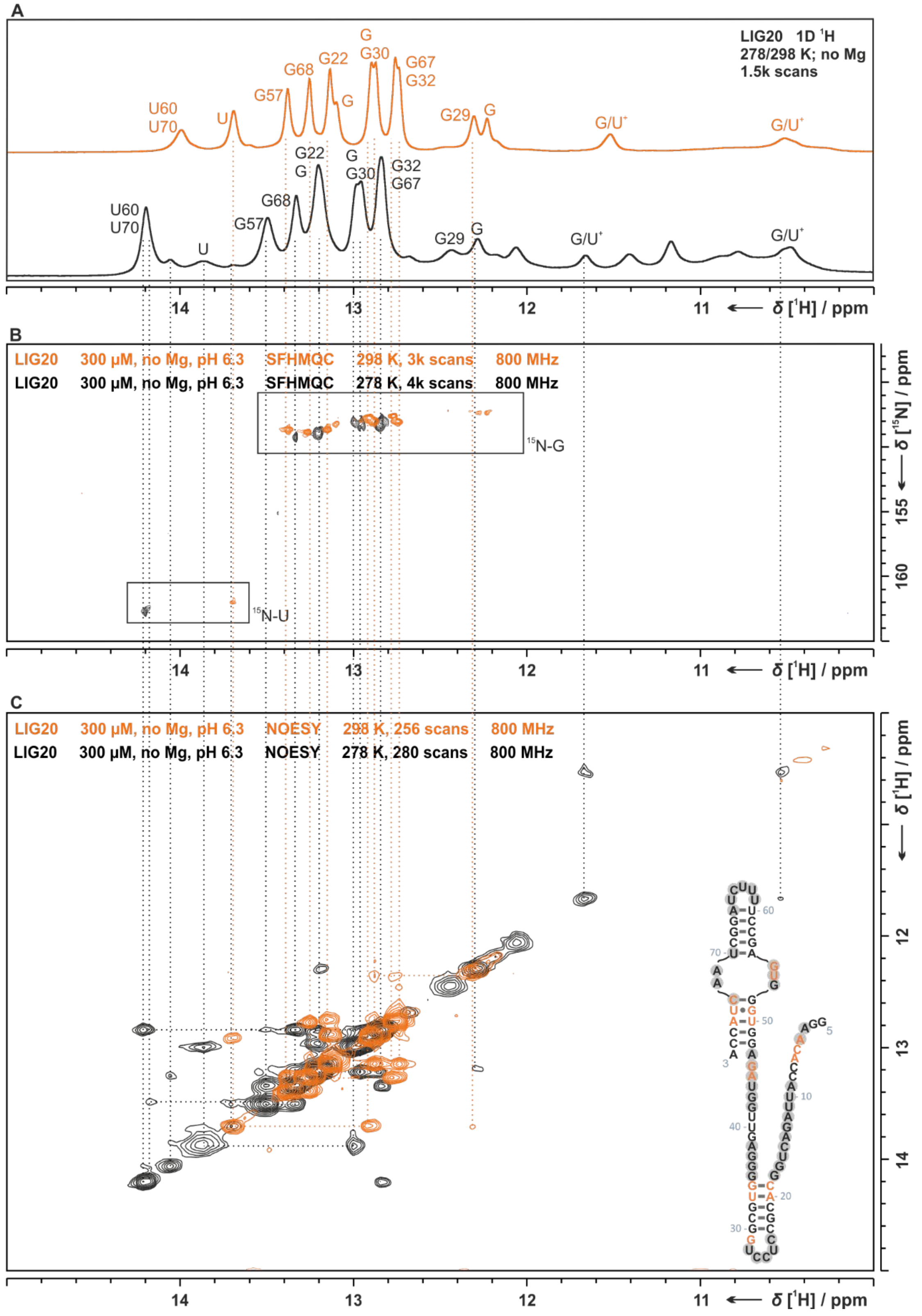

Figure S20 Imino regions of LIG20 at 278 K (black) and at room temperature (orange) NMR spectra. The samples contained as indicated 300 µM RNA with 25 mM potassium phosphate buffer at pH 6.3 with 100 µM NMR reference DSS. Spectra were recorded on Bruker spectrometers. ***A*** *1D ^{1}H spectra recorded with 1.5k at a field of 800 MHz.* ***B*** *2D ^{1}H,^{15}N-SFHMQC spectra recorded with 4k/3k scans at a field of 800 MHz.* ***C*** *2D ^{1}H,^{1}H-NOESY spectra recorded with 280/298 scans and at a field of 800 MHz.*

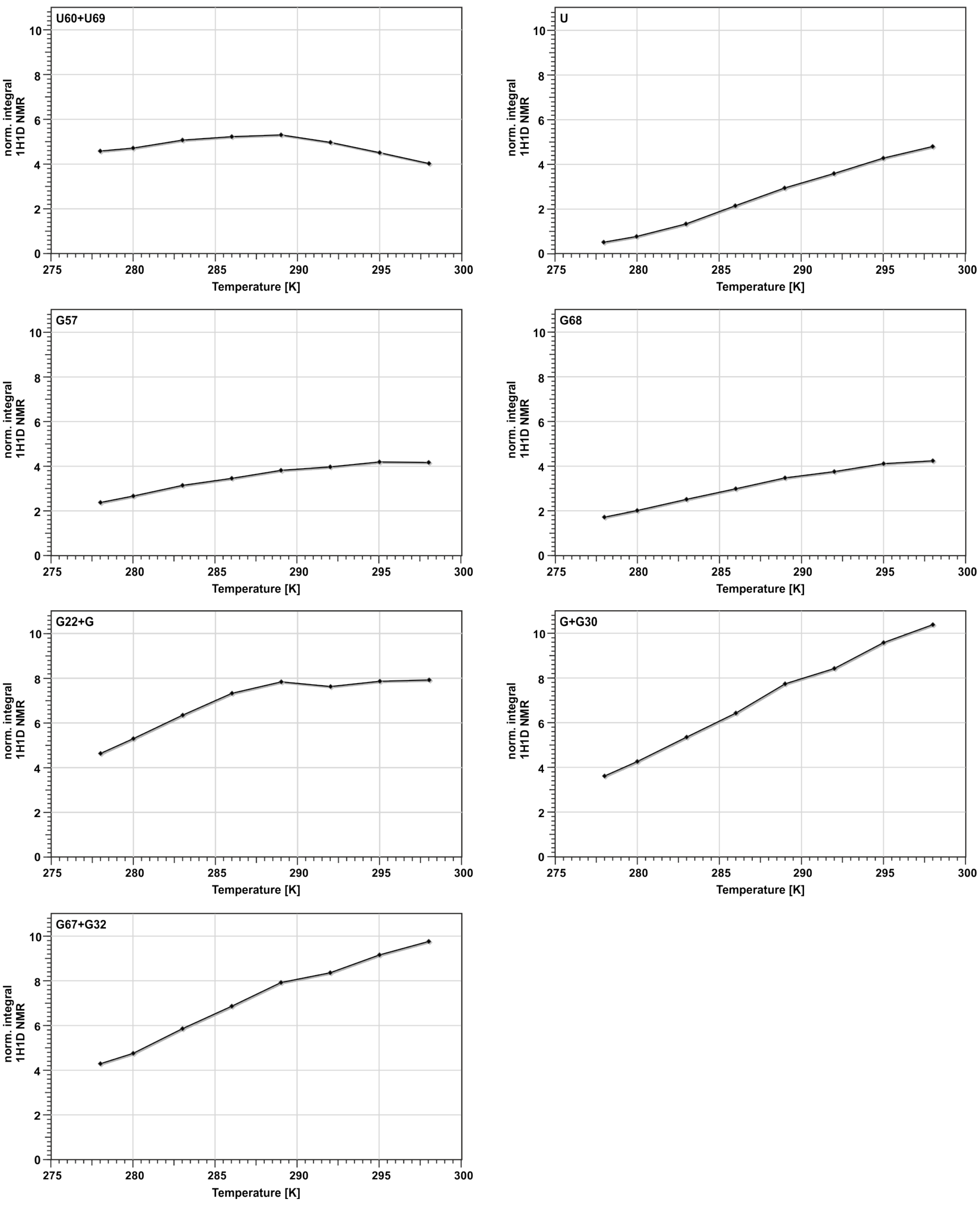

Figure S21 Normalized imino proton signal integral of LIG20 over temperature. The integrals were normalized to the DSS proton signal (9 protons) at 0ppm. The temperature range was 278 K-298 K.

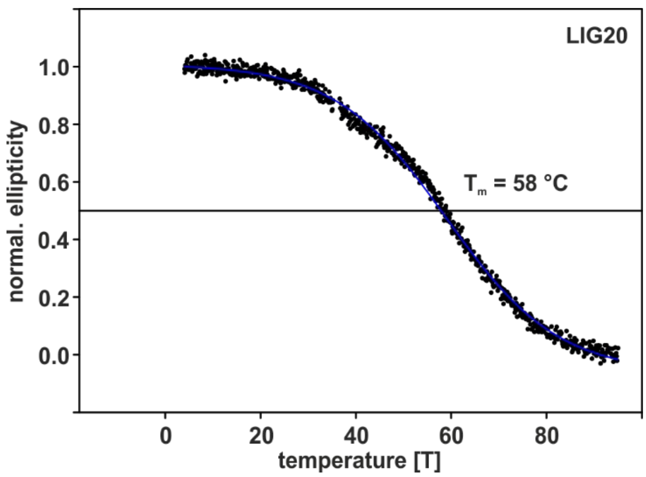

Figure S22 CD melting curve of LIG20 was recorded with a JASCO J-810 spectropolarimeter. The sample contained 100 µM NMR sample of the RNA. Molar ellipticity is shown as a function of temperature. T_m was obtained from a sigmoidal fit.

LIG16 & LIG14

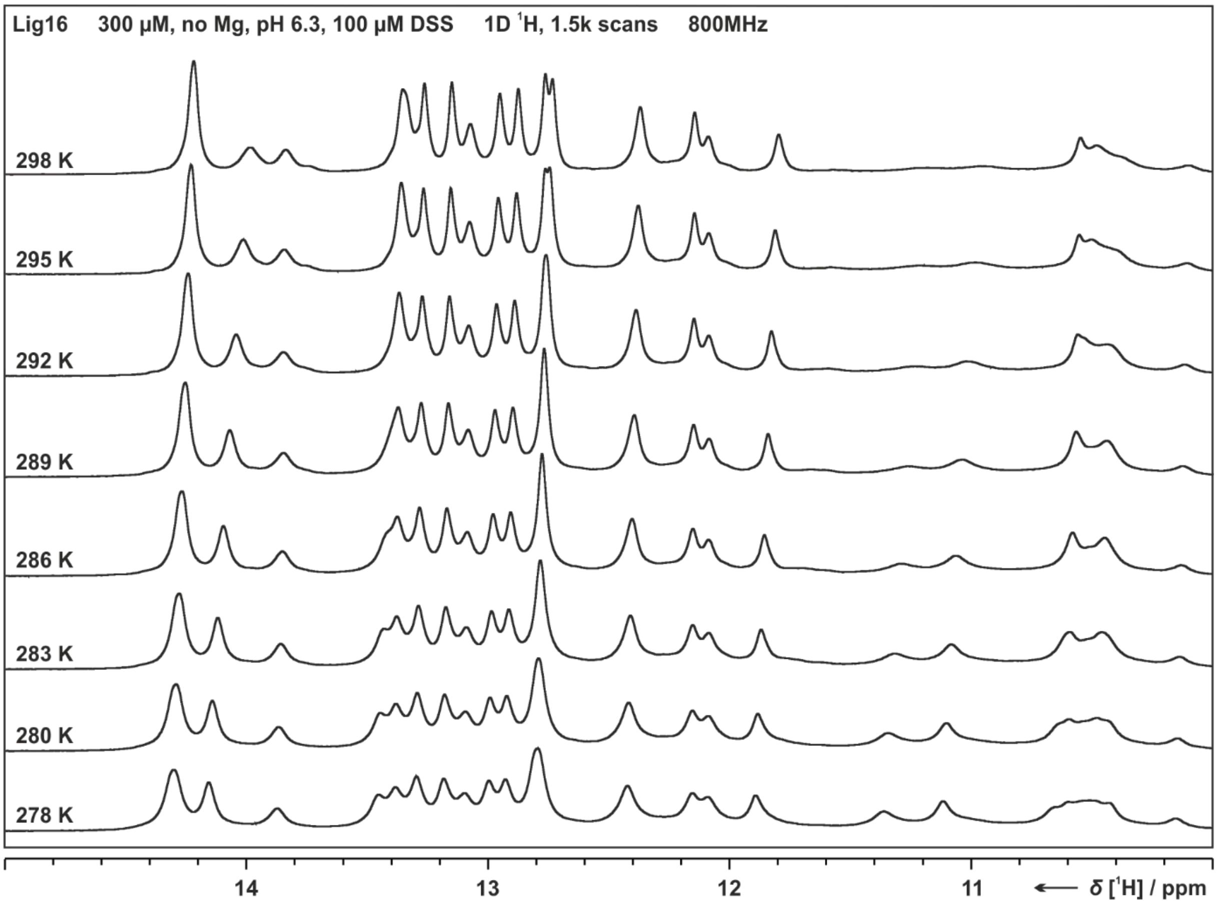

Figure S23 Imino regions 1D ^{1}H NMR spectra of LIG16 at different temperatures ranging from 278 K to 298 K. The spectra were measured with a Bruker spectrometer at 950 MHz with 1.5k scans and a spectral width of 24ppm. The sample contained as indicated 300 µM RNA with 25 mM potassium phosphate buffer (pH 6.3), 100 µM NMR reference DSS and 10% D_2O.

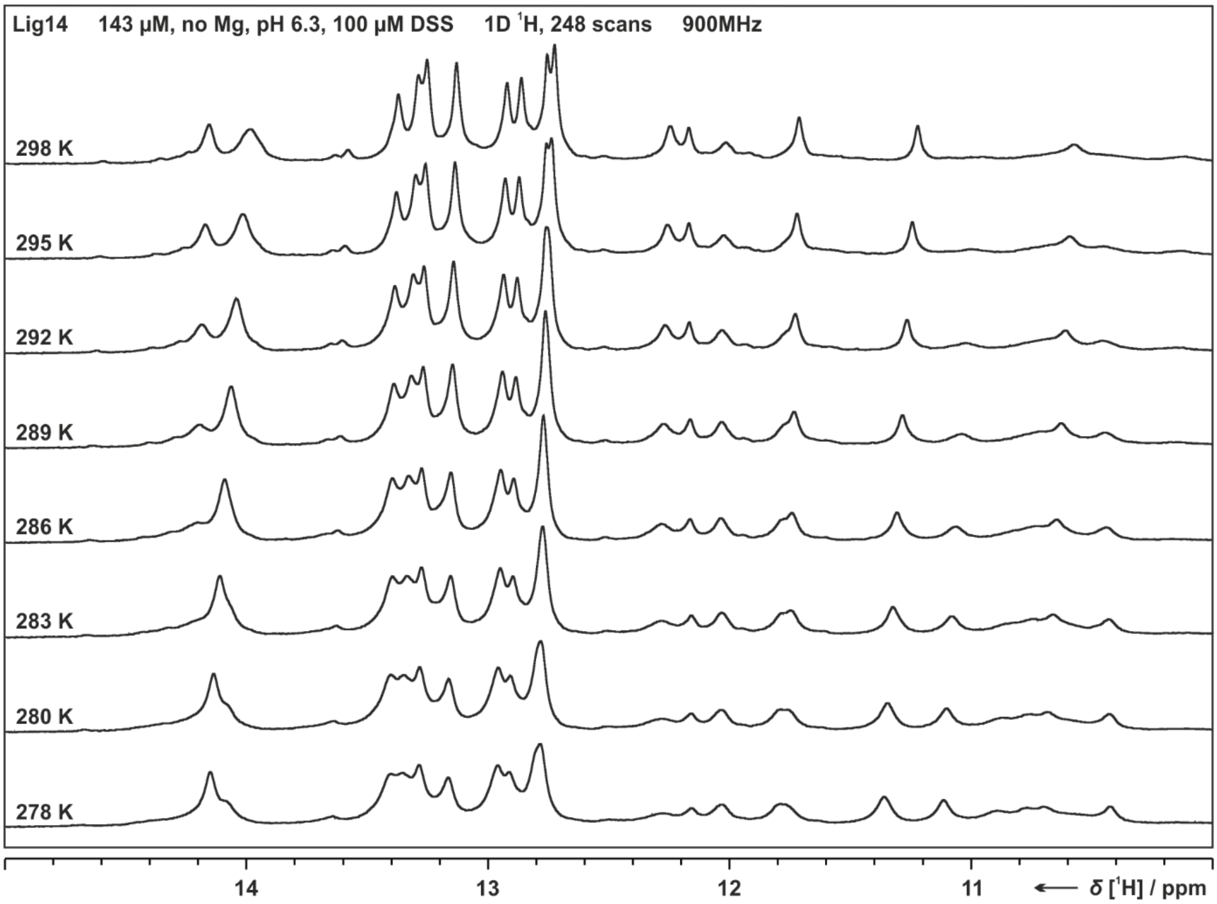

Figure S24 Imino regions 1D ^{1}H NMR spectra of LIG14 at different temperatures ranging from 278 K to 298 K. The spectra were measured with a Bruker spectrometer at 950 MHz with 1.5k scans and a spectral width of 24ppm. The sample contained as indicated 300 µM RNA with 25 mM potassium phosphate buffer (pH 6.3), 100 µM NMR reference DSS and 10% D_2O.

LIG1, INT & HDV1

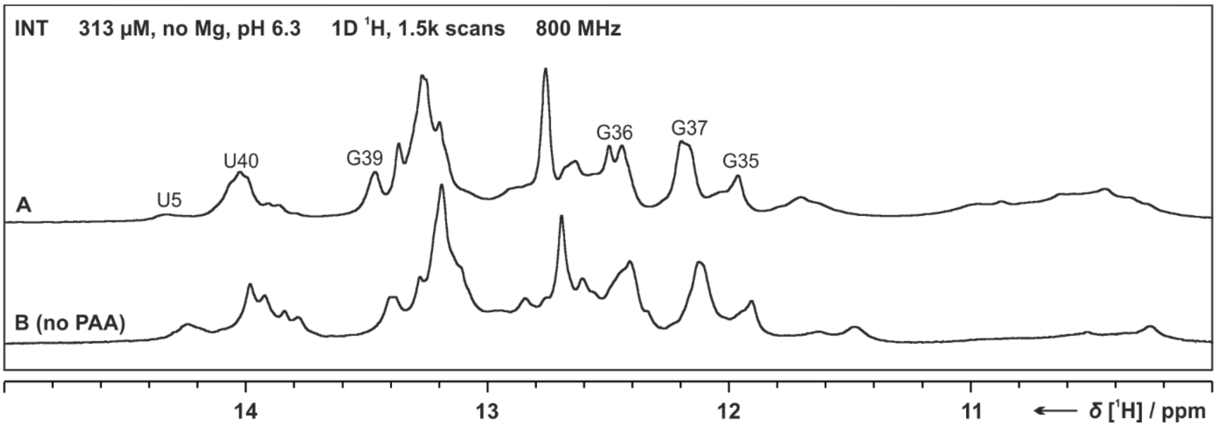

Figure S25 Imino regions 1D ^{1}H NMR spectra of INT. The sample was analyzed first after fast purification with a centrifugal concentrator (B) and it was then purified via preparative PAGE (A). The spectra were measured with a Bruker spectrometer at 298 K and 800 MHz with 1.5k scans and a spectral width of 24ppm. The samples contained as indicated 313 µM RNA with 25 mM potassium phosphate buffer (pH 6.3) and 10% D_2O.

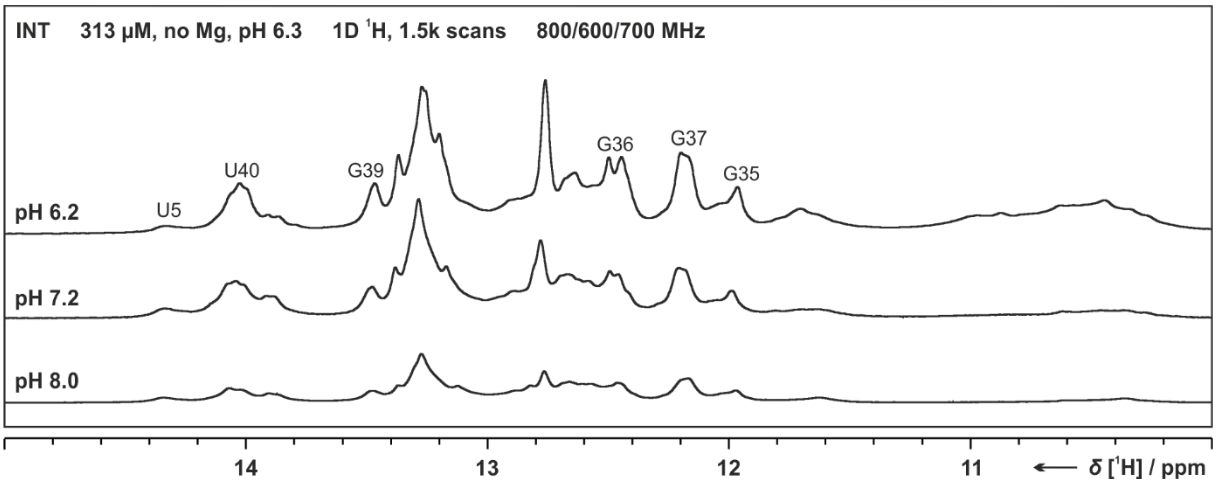

Figure S26 Imino regions 1D ^{1}*H NMR spectra of INT at different pH levels. The pH levels were set by varying buffer composition of* K_2HPO_4*/*KH_2PO_4 *with a concentration of 25 mM. The spectra were measured with a Bruker spectrometer at 298 K and 800 MHz/600 MHz/700 MHz with 1.5k scans and a spectral width of 24ppm. The samples contained as indicated 128 µM RNA with 25 mM potassium phosphate buffer (pH 6.3) and 10%* D_2O*.*

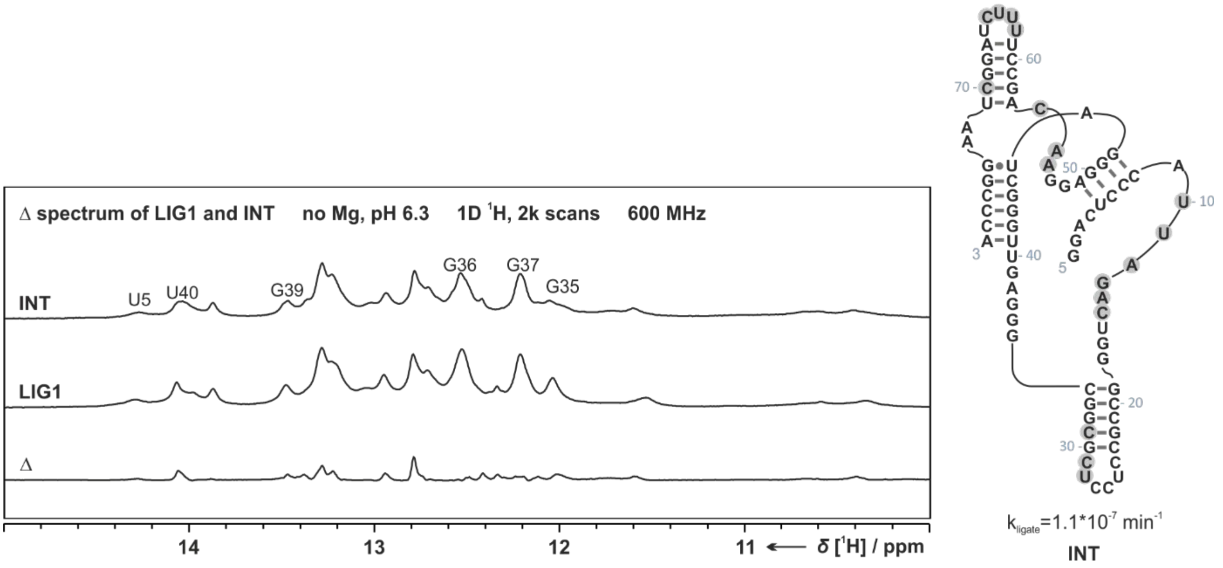

Figure S27 Imino regions 1D ^{1}*H NMR spectra of LIG1 and INT. The spectra were measured with a Bruker spectrometer at 283 K and 600 MHz with 2k scans and a spectral width of 24ppm. The samples contained 300 µM (LIG1) and 134 µM (INT) RNA with 25 mM potassium phosphate buffer (pH 6.3), 100 µM NMR reference DSS and 10%* D_2O*. The signal intensities were normalized. The difference spectrum (Δ) is shown as a calculated power spectrum. The 2*nd *structure of INT is shown. Arrows highlight nucleotides different to INT.*

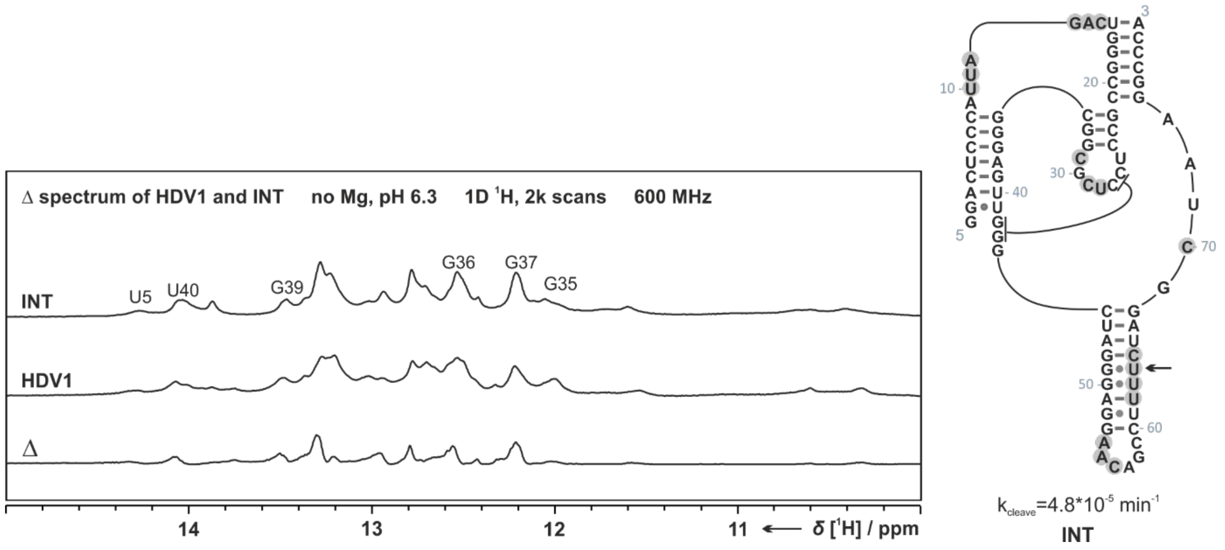

Figure S28 Imino regions 1D ^{1}H NMR spectra of HDV1 and INT. The spectra were measured with a Bruker spectrometer at 283 K and 600 MHz with 2k scans and a spectral width of 24ppm. The samples contained 146 µM (HDV1) and 134 µM (INT) RNA with 25 mM potassium phosphate buffer (pH 6.3), 100 µM NMR reference DSS and 10% D_2O. The signal intensities were normalized. The difference spectrum (Δ) is shown as a calculated power spectrum. The 2nd structure of INT is shown. Arrows highlight nucleotides different to INT.

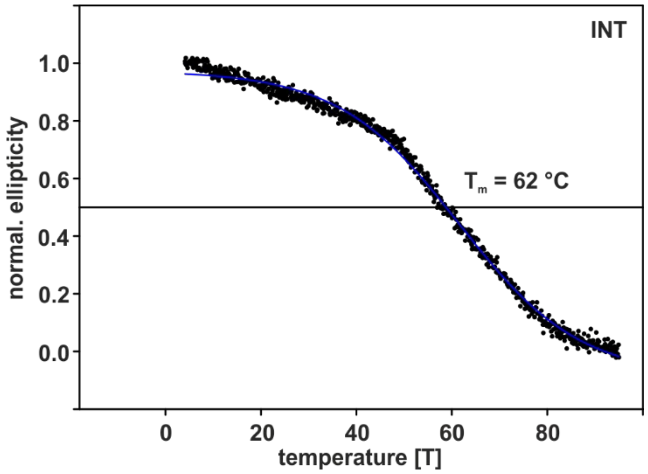

Figure S29 CD melting curve of INT was recorded with a JASCO J-810 spectropolarimeter. The sample contained 100 µM NMR sample of the RNA. Molar ellipticity is shown as a function of temperature. T_m was obtained from a sigmoidal fit.

HDVP

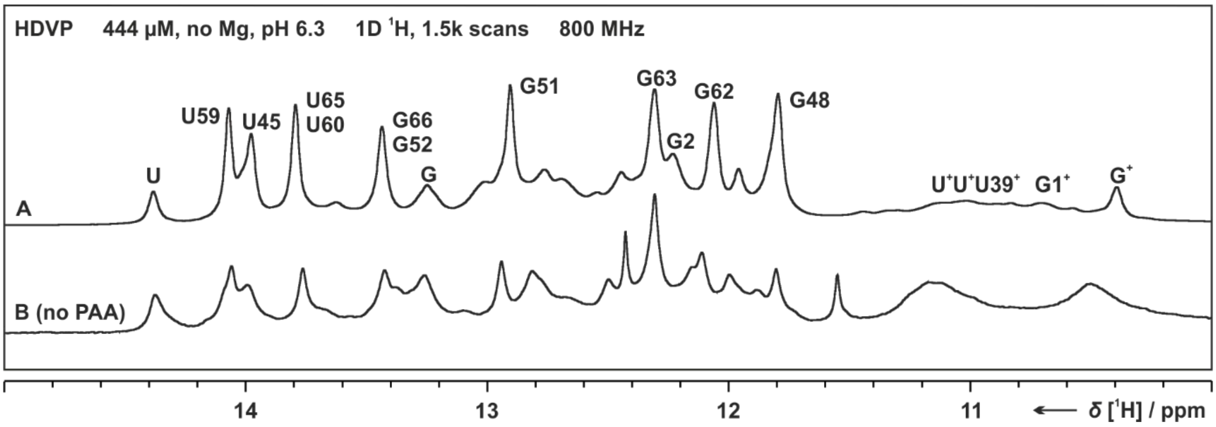

Figure S30 Imino regions 1D ^{1}H NMR spectra of HDVP. The sample was analyzed first after fast purification with a centrifugal concentrator (B) and it was then purified via preparative PAGE (A). Plus signs highlight nucleobase imino protons of non-canonical base pairs. The spectra were measured with a Bruker spectrometer at 298 K and 900 MHz (A)/800 MHz (B) with 1k (A)/3k (B) scans and a spectral width of 24ppm. The samples contained as indicated 444 µM RNA with 25 mM potassium phosphate buffer (pH 6.3), 100 µM NMR reference DSS (A) and 10% D_2O.

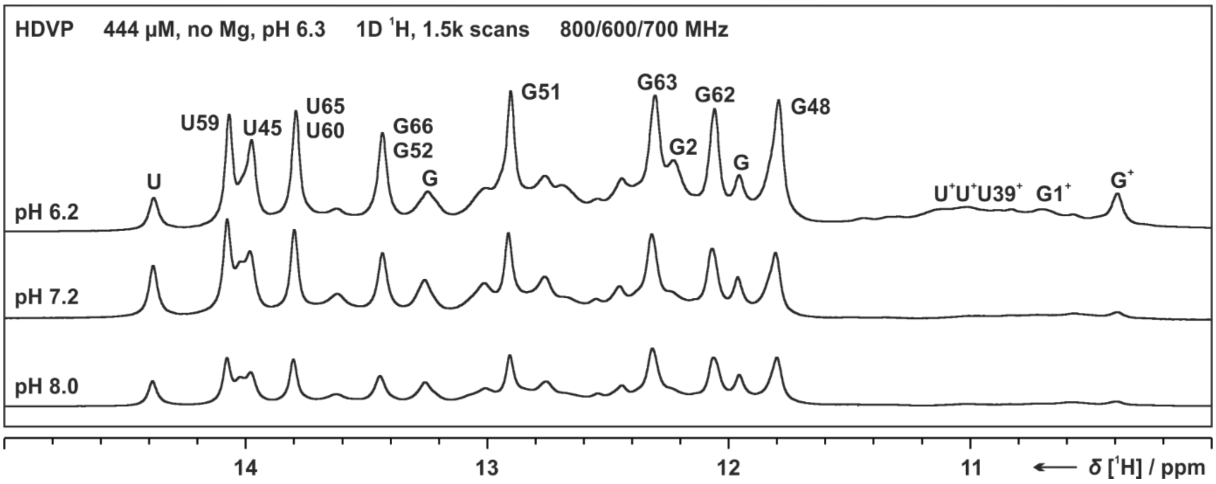

Figure S31 Imino regions 1D ^{1}H NMR spectra of HDVP at different pH levels. The pH levels were set by varying buffer composition of K_2HPO_4/KH_2PO_4 with a concentration of 25 mM. Plus signs highlight nucleobase imino protons of non-canonical base pairs. The spectra were measured with a Bruker spectrometer at 298 K and 800 MHz/600 MHz/700 MHz with 1.5k scans and a spectral width of 24ppm. The samples contained as indicated 444 µM RNA with 25 mM potassium phosphate buffer and 10% D_2O.

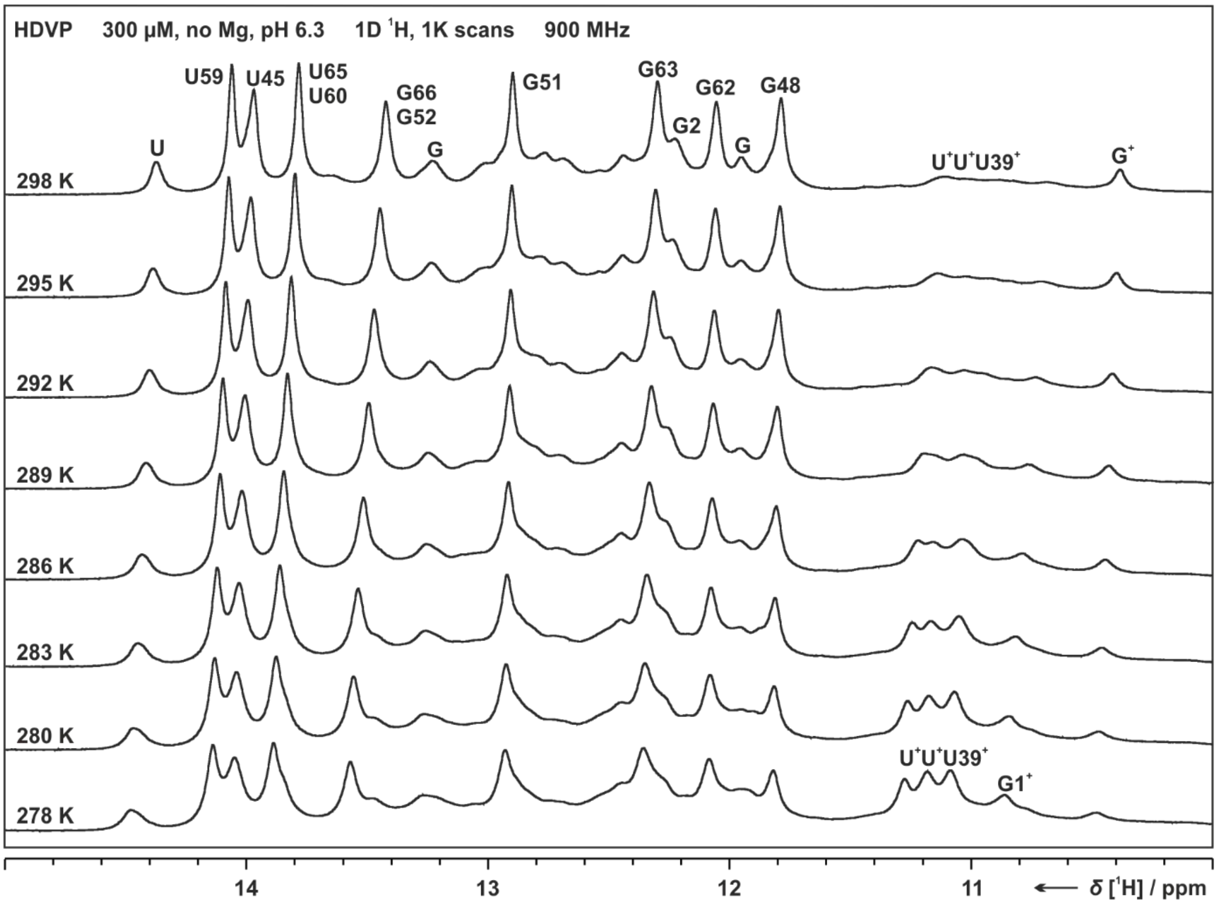

Figure S32 Imino regions 1D ^{1}H NMR spectra of HDVP at different temperatures ranging from 278 K to 298 K. Plus signs highlight nucleobase imino protons of non-canonical base pairs. The spectra were measured with a Bruker spectrometer at 900 MHz with 1k scans and a spectral width of 24ppm. The samples contained as indicated 300 µM RNA with 25 mM potassium phosphate buffer (pH 6.3), 100 µM NMR reference DSS and 10% D_2O.

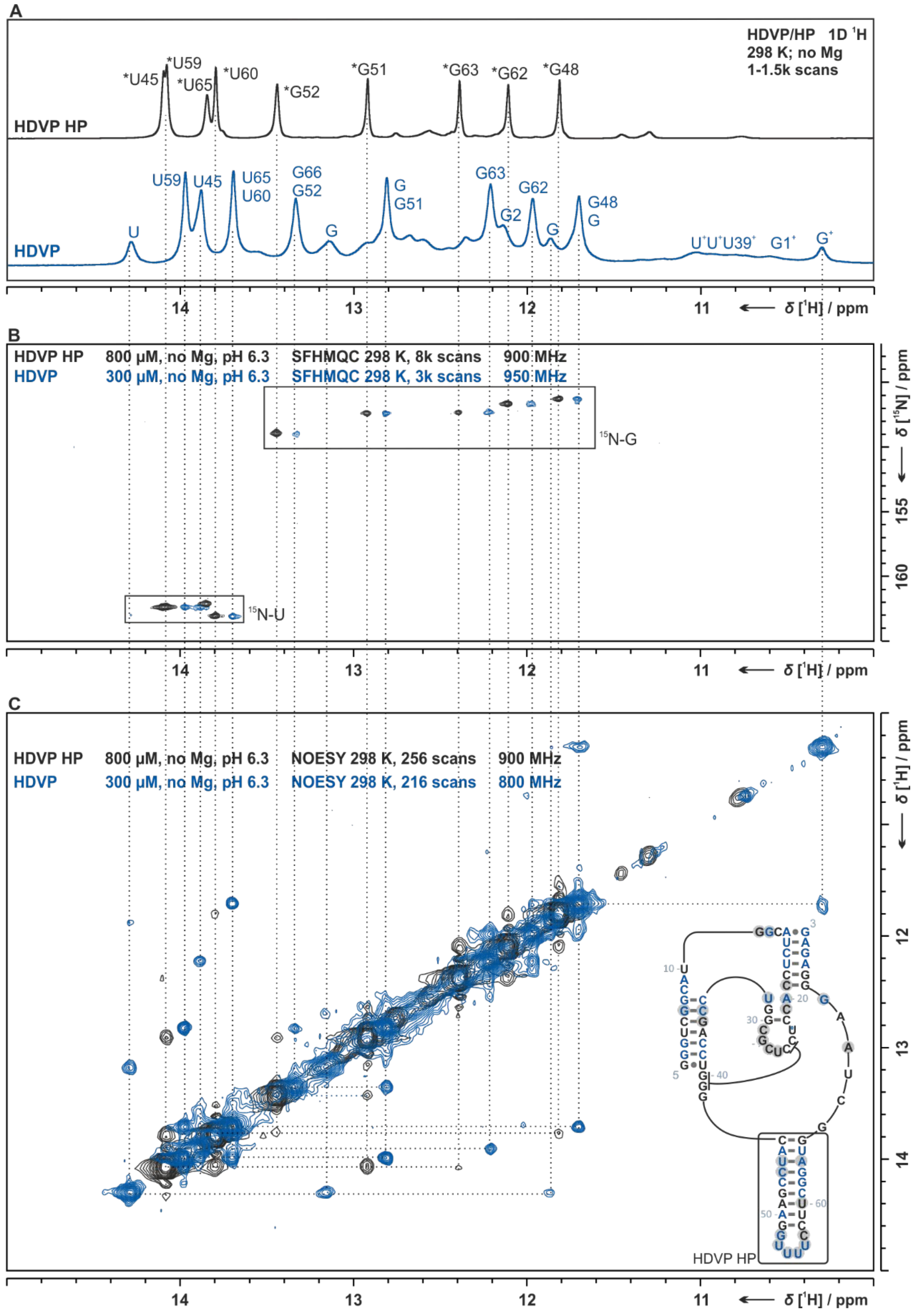

Figure S33 Imino regions of HDVP (blue) and HDVP HP (black) NMR spectra at room temperature (298K). The samples contained 800 µM (HDVP HP) and 300 µM (HDVP) RNA with 25 mM potassium phosphate buffer at pH 6.3 with 100 µM NMR reference DSS. Plus signs highlight nucleobase imino protons of non-canonical base pairs. Spectra were recorded on Bruker spectrometers. ***A*** *1D ^{1}H spectrum recorded with 1.5k/1k scans at 800/900 MHz.* ***B*** *2D $^{1}H,^{15}N$-SFHMQC spectrum recorded with 8k/3k scans at 900/950 MHz.* ***C*** *2D $^{1}H,^{1}H$-NOESY spectrum recorded with 256/216 scans at 900/800 MHz.*

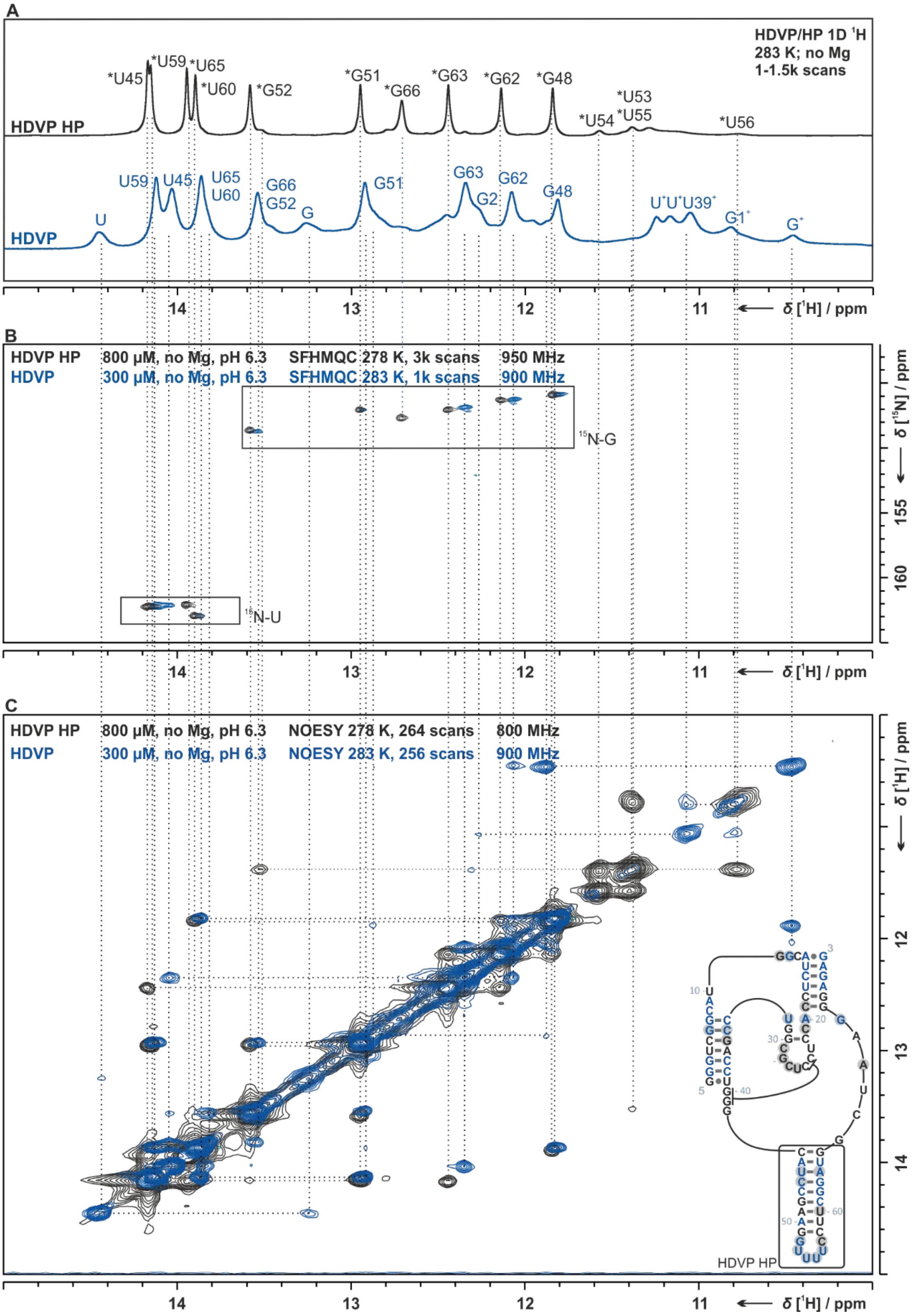

Figure S34 Imino regions of HDVP (blue) and HDVP HP (black) NMR spectra at low temperature. The samples conatined 1200 µM (HDVP HP) and 700 µM (HDVP) RNA with 25 mM potassium phosphate buffer at pH 6.3 with 100 µM NMR reference DSS. Plus signs highlight nucleobase imino protons of non-canonical base pairs. Spectra were recorded at Bruker spectrometers. ***A*** *1D ^{1}H spectrum recorded with 1.5k/1k scans at 278/283 K and 800/900 MHz.* ***B*** *2D $^{1}H,^{15}N$-SFHMQC spectrum recorded with 3k/1k scans at 278/283 K and 950/900 MHz.* ***C*** *2D $^{1}H,^{1}H$-NOESY recorded measured with 264/256 scans at 278/283 K and 800/900 MHz.*

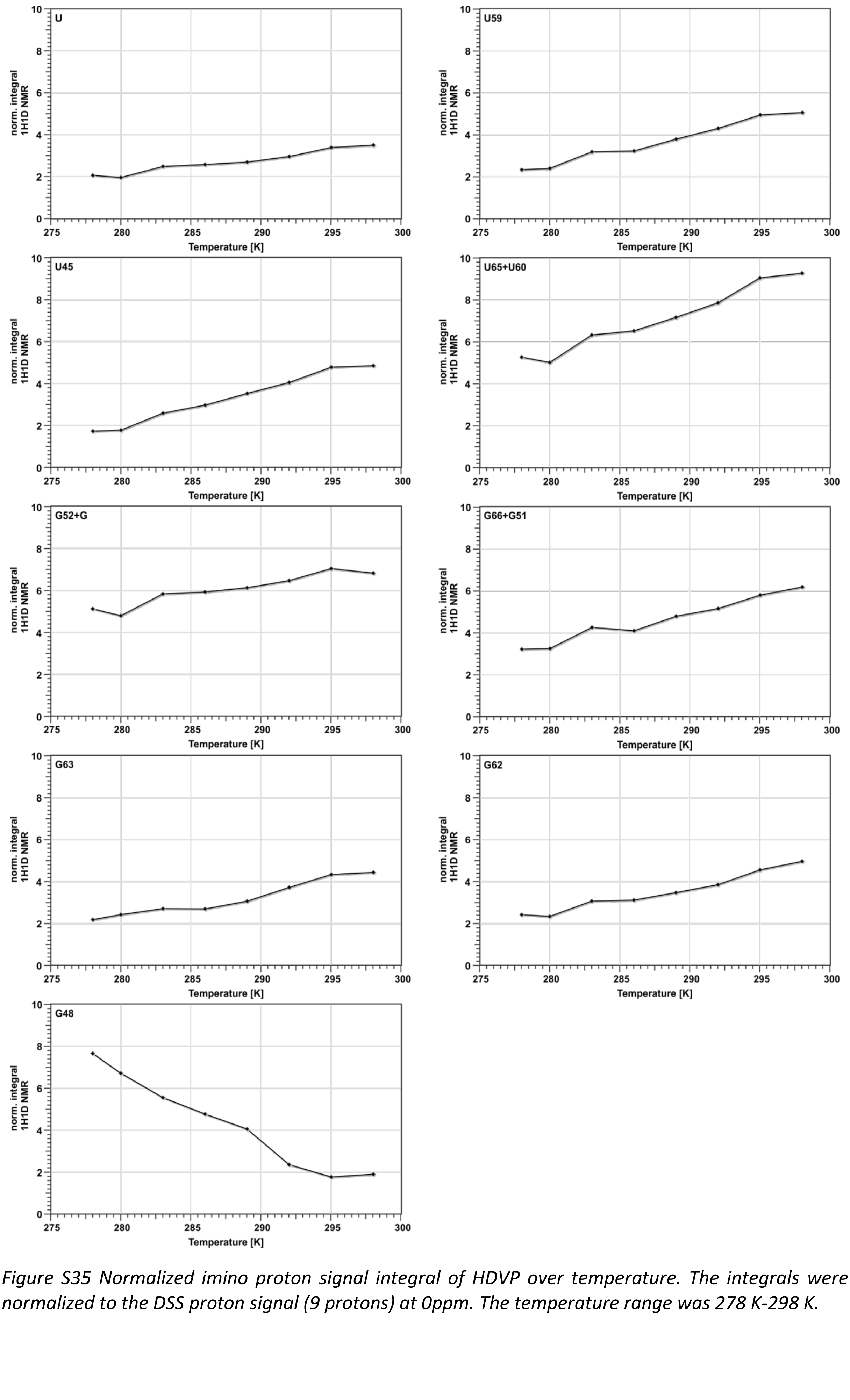

Figure S35 Normalized imino proton signal integral of HDVP over temperature. The integrals were normalized to the DSS proton signal (9 protons) at 0ppm. The temperature range was 278 K-298 K.

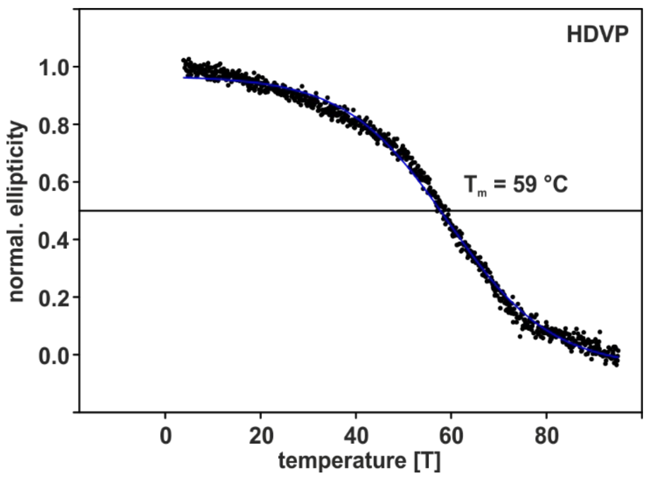

Figure S36 CD melting curve of HDVP was recorded with a JASCO J-810 spectropolarimeter. The sample contained 100 µM NMR sample of the RNA. Molar ellipticity is shown as a function of temperature. T_m was obtained from a sigmoidal fit.

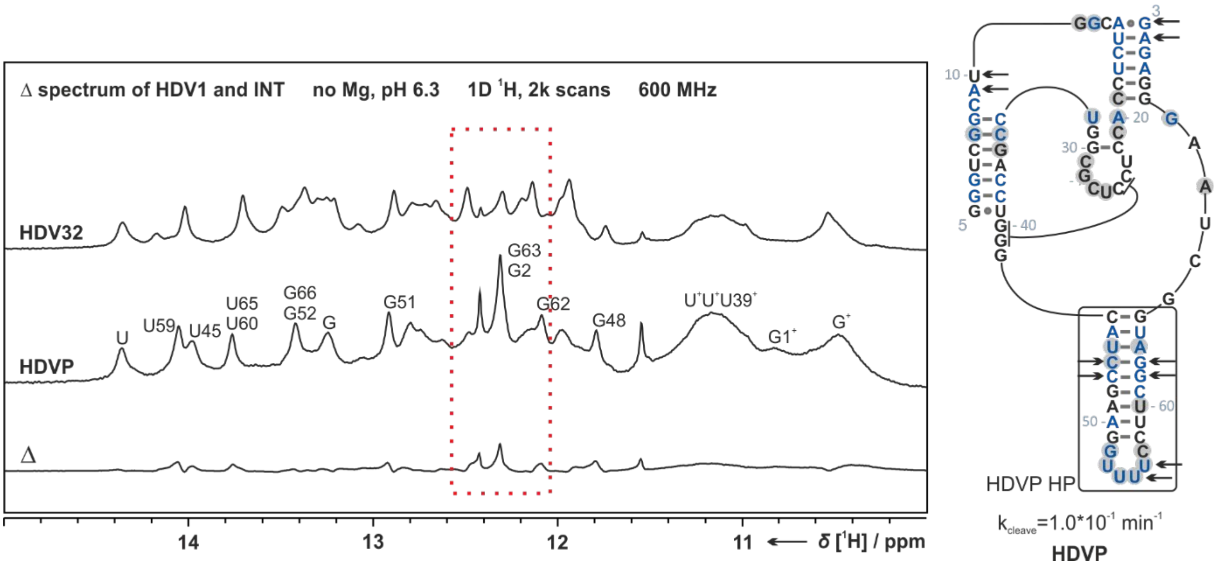

Figure S37 Imino regions 1D ^{1}H NMR spectra of HDVP and HDV32. Plus signs highlight nucleobase imino protons of non-canonical base pairs. The spectra were measured with a Bruker spectrometer at 283 K and 600 MHz with 2k scans and a spectral width of 24ppm. The samples contained 100 µM (HDVP) and 300 µM (HDV32) RNA with 25 mM potassium phosphate buffer (pH 6.3), 100 µM NMR reference DSS and 10% D_2O. The signal intensities were normalized. The difference spectrum (Δ) is shown as a calculated power spectrum. The red dashed box indicates differences in the spectra by comparison. The black dashed box shows an artefact. The 2nd structure of LIGP is shown. Arrows highlight nucleotides different to LIG36.

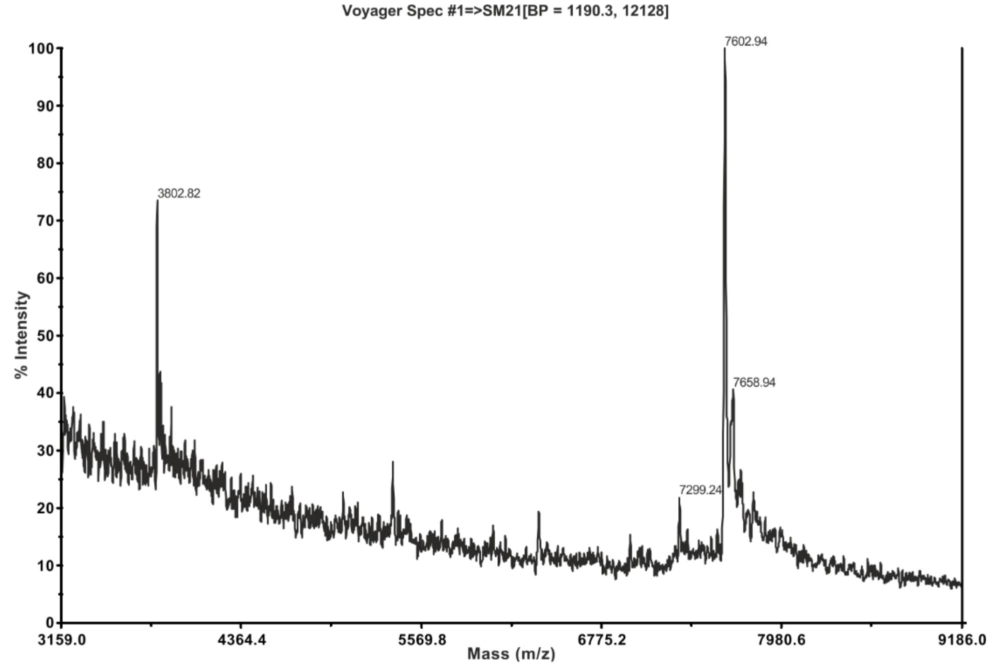

Figure S38 Mass spectrum of HDVP HP. It was recorded through Voyager-DE STR from Applied Biosystems (MALDI). The calculated mass for the 24 nt RNA was 7606.5 u (difference possibly caused by deprotonation, fragmentation or a matrix problem seen before).

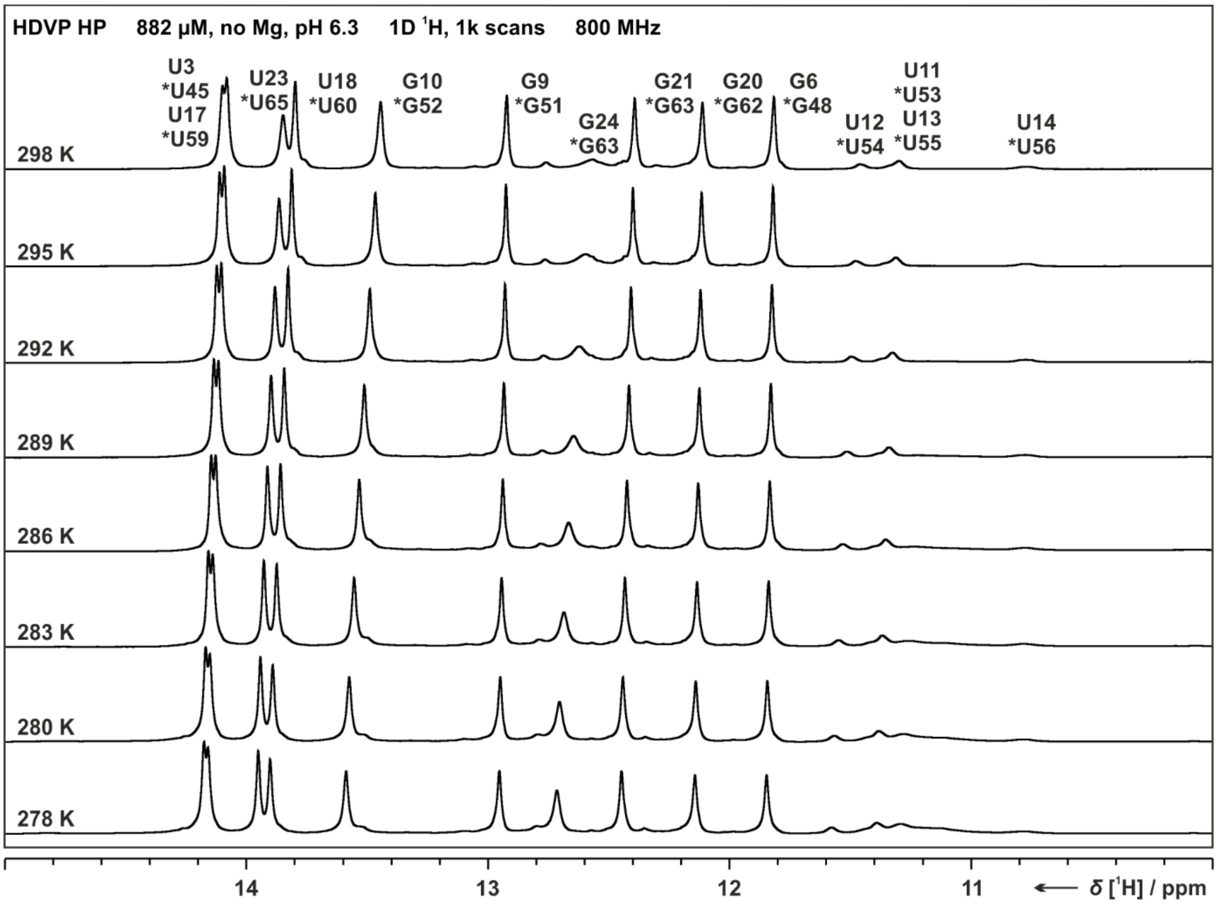

Figure S39 Imino regions 1D ^{1}H NMR spectra of HDVP HP at different temperatures ranging from 278 K to 298 K. Stars indicate nucleotide nomenclature in accordance with the full length construct HDVP. The spectra were measured with a Bruker spectrometer at 800 MHz with 1.5k scans and a spectral

width of 24ppm. The samples contained as indicated 882 µM RNA with 25 mM potassium phosphate buffer (pH 6.3), 100 µM NMR reference DSS and 10% D_2O.

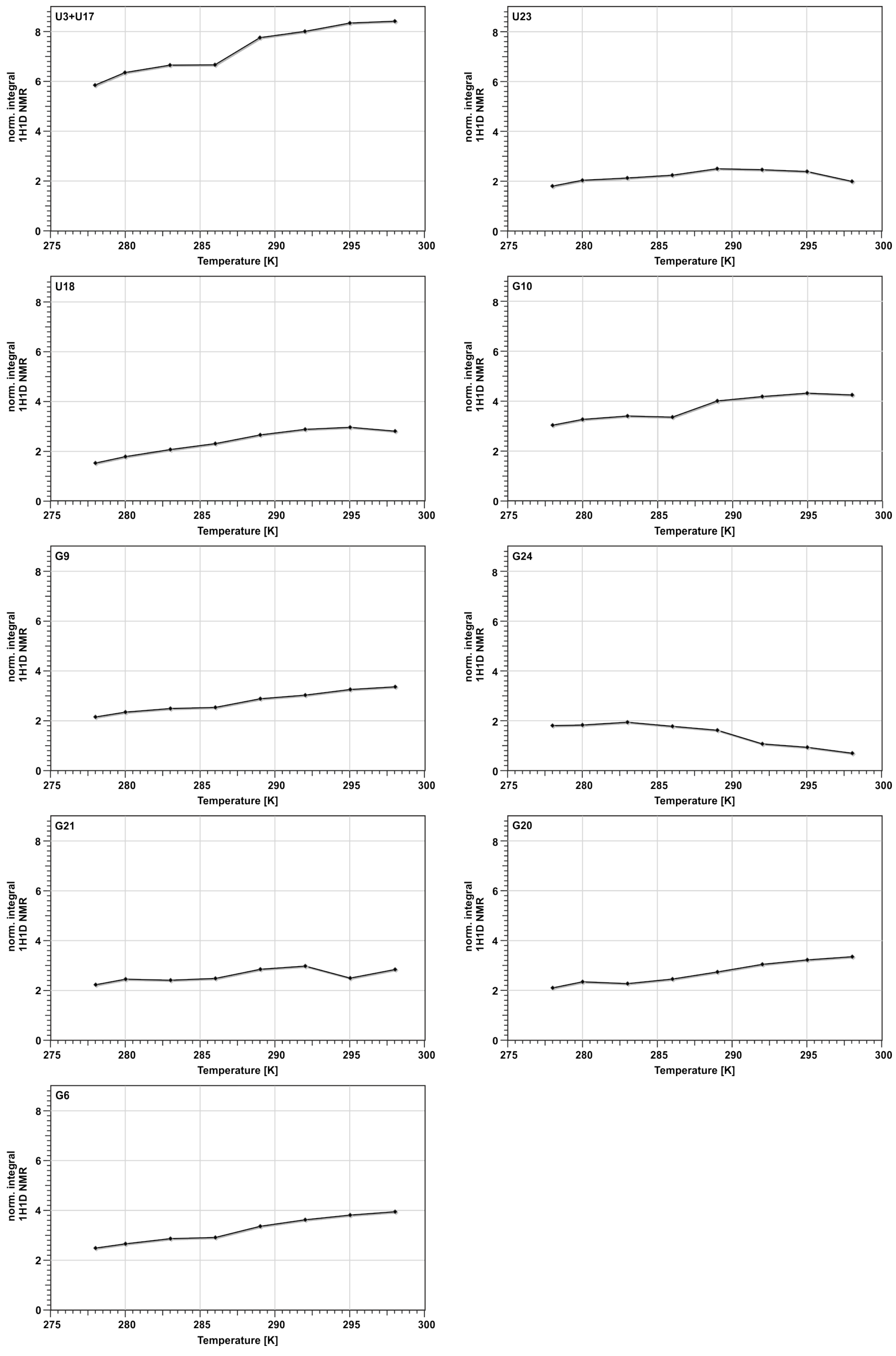

Figure S40 Normalized imino proton signal integral of HDVP HP over temperature. The integrals were normalized to the DSS proton signal (9 protons) at 0ppm. The temperature range was 278 K-298 K.

[1] P. Cordero, W. Kladwang, C. C. VanLang, R. Das, in *Methods Mol. Biol.*, **2015**, pp. 53–77.

[2] S. Yoon, J. Kim, J. Hum, H. Kim, S. Park, W. Kladwang, R. Das, *Bioinformatics* **2011**, *27*, 1798–1805.
